# Age-related penetrance of the *C9orf72* repeat expansion

**DOI:** 10.1038/s41598-017-02364-1

**Published:** 2017-05-18

**Authors:** Natalie A. Murphy, Karissa C. Arthur, Pentti J. Tienari, Henry Houlden, Adriano Chiò, Bryan J. Traynor

**Affiliations:** 1 Neuromuscular Diseases Research Section, Laboratory of Neurogenetics, National Institute on Aging, National Institutes of Health, Bethesda, Maryland 20892 USA; 2 Geisinger Commonwealth School of Medicine, Scranton, Pennsylvania 18509 USA; 3 Department of Neurology, Helsinki University Hospital, Helsinki, FIN-02900 Finland; 4 Molecular Neurology Research Program Unit, University of Helsinki, Helsinki, FIN-02900 Finland; 5 Department of Molecular Neurosciences, Institute of Neurology, University College London, Queen Square House, London, WC1N 3BG UK; 6 Department of Neuroscience, University of Turin, 10126 Turin, Italy; 7 Brain Science Institute, Department of Neurology, Johns Hopkins University, Baltimore, Maryland 21205 USA

## Abstract

A pathogenic hexanucleotide repeat expansion within the *C9orf72* gene has been identified as the major cause of two neurodegenerative syndromes, amyotrophic lateral sclerosis (ALS) and frontotemporal dementia (FTD). This mutation is known to have incomplete penetrance, with some patients developing disease in their twenties and a small portion of carriers surviving to their ninth decade without developing symptoms. Describing penetrance by age among *C9orf72* carriers and identifying parameters that alter onset age are essential to better understanding this locus and to enhance predictive counseling. To do so, data from 1,170 individuals were used to model penetrance. Our analysis showed that the penetrance was incomplete and age-dependent. Additionally, familial and sporadic penetrance did not significantly differ from one another; ALS cases exhibited earlier age of onset than FTD cases; and individuals with spinal-onset exhibited earlier age of onset than those with bulbar-onset. The older age of onset among female cases in general, and among female bulbar-onset cases in particular, was the most striking finding, and there may be an environmental, lifestyle, or hormonal factor that is influencing these penetrance patterns. These results will have important applications for future clinical research, the identification of disease modifiers, and genetic counseling.

## Introduction

Amyotrophic lateral sclerosis (ALS) is a neurodegenerative disease characterized by rapidly progressive paralysis and death due to respiratory failure, typically within 2–4 years of onset. Approximately 7,000 Americans die annually from ALS, making it the most common adult-onset motor neuron disease^1^. The number of ALS cases across the globe will increase to nearly 400,000 in 2040, predominantly due to aging of the population^2^. Furthermore, the annual cost per patient with ALS, as well as the burden placed on family and caregivers, is among the highest for any neurological disease^3^.

The discovery of the pathogenic hexanucleotide repeat expansion in *C9orf72* was a major advance in our understanding of the etiology of ALS and frontotemporal dementia (FTD)^4, 5^. The *C9orf72* repeat expansion accounts for 1 in 10 of every ALS case among European-ancestry populations^5, 6^. This represents the first time that a large non-coding repeat expansion has been implicated in either of these neurodegenerative diseases. There is a growing sense of optimism that antisense oligonucleotide therapy or a small molecule therapeutic may be effective in slowing progression in patients carrying the expansion, and such gene therapy trials may commence enrollment in the near future^7^.

The variable phenotype associated with this mutation complicates these clinical trial efforts, especially future studies testing the ability of therapies to delay disease onset among asymptomatic carriers. For example, the age of symptom onset fluctuates widely among mutation carries, ranging from 40 to 90 years of age. Some carriers do not manifest disease even in their ninth decade of life, indicating that penetrance of this mutation is incomplete. We previously published age-related estimates of *C9orf72* penetrance based on a relatively small cohort of carriers^6^. Here, we aim to improve on these estimates by incorporating a larger cohort. We also investigate which clinical and demographic factors influence penetrance. A better understanding of the age-related penetrance of the *C9orf72* repeat expansion will help in clinical trial design, and ultimately in determining at what stage to initiate neuroprotective therapy, should it become available in the future.

## Results

Penetrance is the proportion of individuals carrying a mutated gene that also express the affected phenotype^8^. Age-related penetrance indicates that the expression of the phenotype in mutant-gene carriers is dependent upon the individual’s age. To gather the necessary raw data to calculate penetrance of the *C9orf72* mutation, we performed a literature search in PubMed.

**Figure 1:**
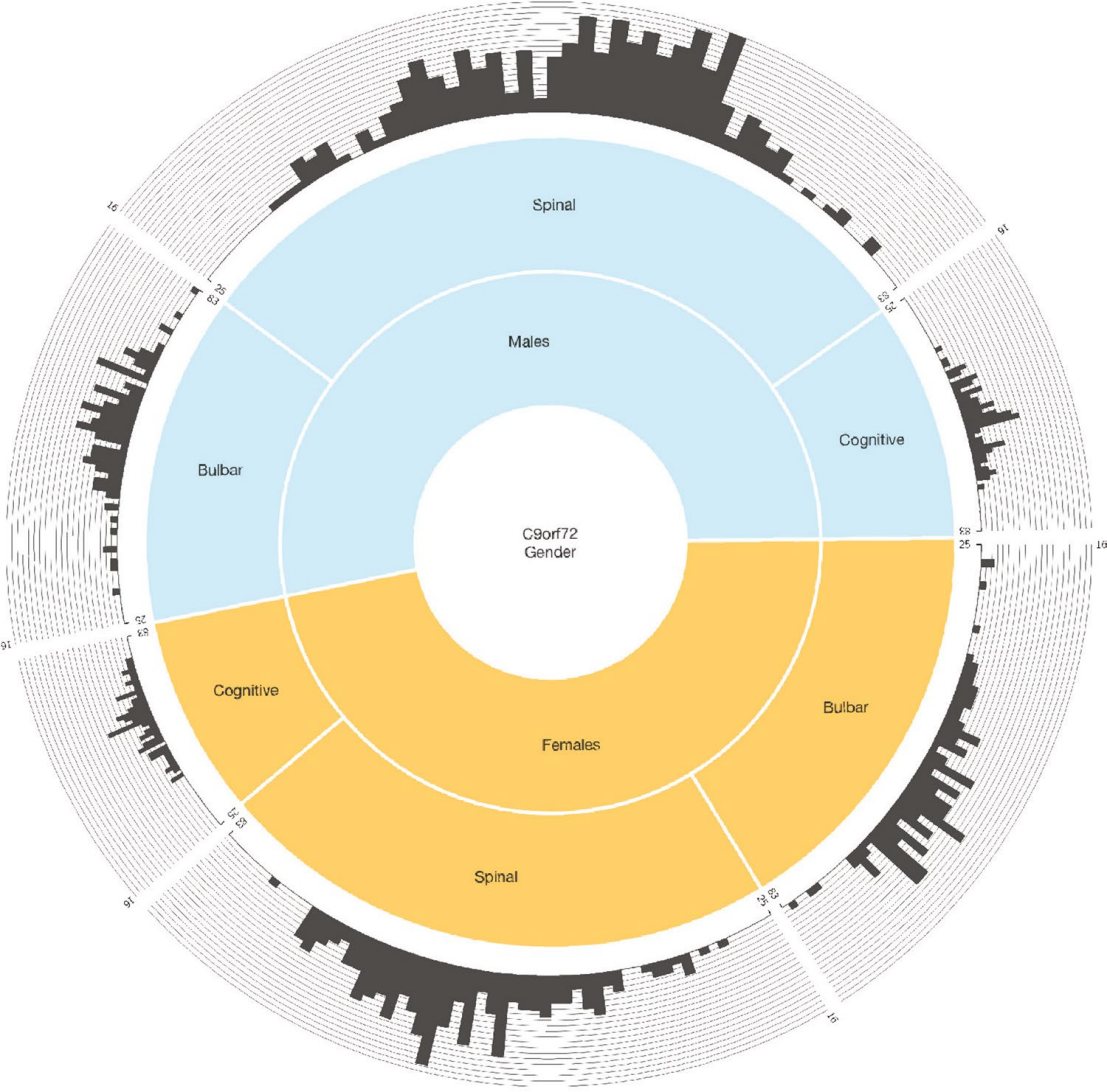
Relationship between gender and site of onset in *C9orf72* repeat expansion carriers. Donut chart showing the site of symptom onset and age at onset among male and female *C9orf72* carriers. Inner layer shows the proportion of males (52.6%) and females (47.4%) in the cohort. The middle layer shows the site of onset within males and females. The outer histogram layer shows the age distribution within each subset. The y-axis of this outer histogram represents the number of patients at each age, whereas the x-axis represents age at symptom onset ranging from 25 to 83 years of age. The graph was created using *Circos* (version 0.67, available at www.circos.ca) and *FusionCharts* (www.jsfiddle.net/fusioncharts/J7svz).

Our literature search identified 37,991 patients diagnosed with neurodegenerative disease who were screened for the presence of the *C9orf72* repeat expansion. Of these, 3,457 carried the pathogenic mutation, representing a carrier rate of 9.1% among cases. After excluding those with insufficient data, 1,147 cases of repeat expansion were available for analysis based on a cohort of 13,672 (8.4%). The majority of these cases presented with ALS (n = 735, 64.0% of the total). Out of 36,420 total control samples that were reported in the literature, 40 had the repeat expansion (0.1%). This number was reduced to 23 after excluding those with unavailable age of onset data (0.06%). In summary, data from 1,170 individuals was available for analysis. The complete data set for the 1,170 individuals can be found as Supplemental Table S2, and the gender, site of onset, and age at onset distributions of this cohort are shown in Fig. 1.

**Figure 2:**
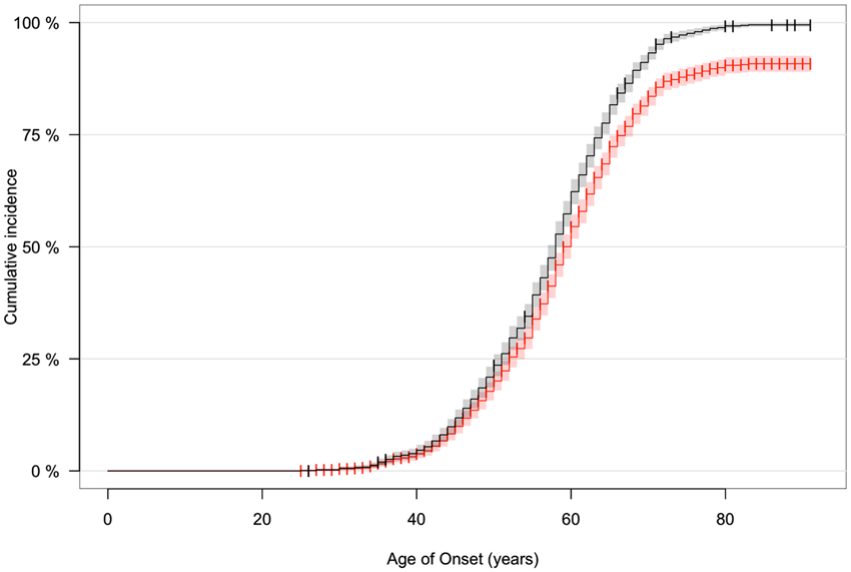
Age-related penetrance of the pathogenic *C9orf72* repeat expansion. The black curve shows age-related penetrance of 1,147 symptomatic patients and 23 asymptomatic individuals carrying the *C9orf72* repeat expansion. The shading represents the 95% confidence interval and censored individuals (i.e. asymptomatic) are indicated by crosses. The red curve represents model data in which the proportion of asymptomatic individuals is artificially increased to 20% (n = 316).

Our data showed that median age at symptom onset among carriers of the *C9orf72* repeat expansion was 58.0 years of age (95% confidence interval: 57.0 to 59.0; see black curve in Fig. 2). The youngest reported age at symptom onset was 25 years among a man who presented with ALS. Penetrance was cumulatively nearly complete (99.5%) by 83 years of age. More than half (n = 13, 56.5%) of the neurologically normal individuals carrying the repeat expansion were younger than the median age of onset.

The selection of data based on random cohorts of screened patients and controls may be subject to ascertainment bias compared to estimates based on selection within families. This bias arises from a decreased enrollment among asymptomatic carriers. To model this possibility, we artificially increased our percentage of control subjects from 2.0% to 20% by adding an additional 293 asymptomatic subjects and randomly assigning them ages at screening that ranged from the youngest to the oldest ages at onset observed among our cases (25 to 91 years of age). The modeled data and the actual data had a statistically significant difference (median age of symptom onset = 60.0 years of age; Log-rank test = 66.2, p-value = 4.4 × 10^−16^; see red curve in Fig. 2). Notably, penetrance at older ages was lower when the control rate was increased (90.9% at age 83). More data on very elderly asymptomatic carriers is needed to precisely estimate the cumulative penetrance at old age. We used our original data with a 2.0% control rate in our subsequent analysis of penetrance, keeping in mind that the cumulative penetrance in the oldest age-groups may be less accurate.

**Figure 3:**
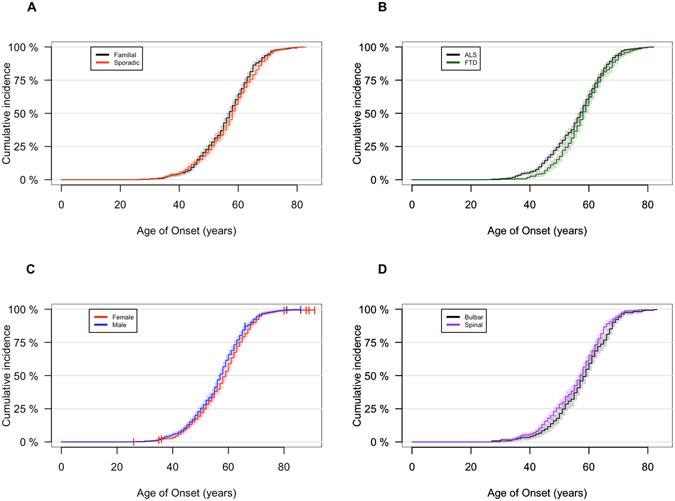
Age-related penetrance of the pathogenic *C9orf72* repeat expansion classified by subtype. (**A**) Age-related penetrance based on familial (n = 555) and sporadic (n = 544) status; (**B**) age-related penetrance curves based on ALS (n = 734) and FTD (n = 303) clinical phenotype; (**C**) age-related penetrance curves comparing male (n = 612) and female (n = 543) cases of ALS and FTD; (**D**) age-related penetrance curves among ALS patients based on bulbar (n = 270) or spinal (n = 481) site of symptom onset. The shading represents the 95% confidence interval and censored individuals (i.e. asymptomatic) are indicated by crosses.

We next explored clinical and demographic factors that may influence age-related penetrance among *C9orf72* carriers. We observed that familial and sporadic *C9orf72* cases had nearly identical penetrance by age (median age of onset among familial *C9orf72* cases = 57.0 years of age, 95% confidence interval (CI): 56.0–58.0; median age of onset among sporadic *C9orf72* cases = 58.0, 95% CI: 58.0–59.0; Log-rank test = 3.2, p-value = 0.07; see Fig. 3A).

Penetrance by age was modestly higher among *C9orf72* carriers presenting with pure ALS (median age of onset = 57.0 years of age, 95% CI: 56.0–58.0) compared to those presenting with pure FTD (median age of onset = 58.0 years of age, 95% CI: 57.0–60.0; Log-rank test = 4.4, p-value = 0.04; see Fig. 3B).

Male carriers of *C9orf72* had a greater likelihood of developing disease at a younger age than females (see Fig. 3C). Though this difference was relatively small (2-year difference in median survival), it was observed over nearly the entire range of age at onset and was statistically significant (median age of onset in males = 57.0 years of age, 95% CI: 56.0–58.0; median age of onset in females = 59.0, 95% CI: 58.0–60.0; Log-rank test = 5.1, p-value = 0.02).

**Figure 4:**
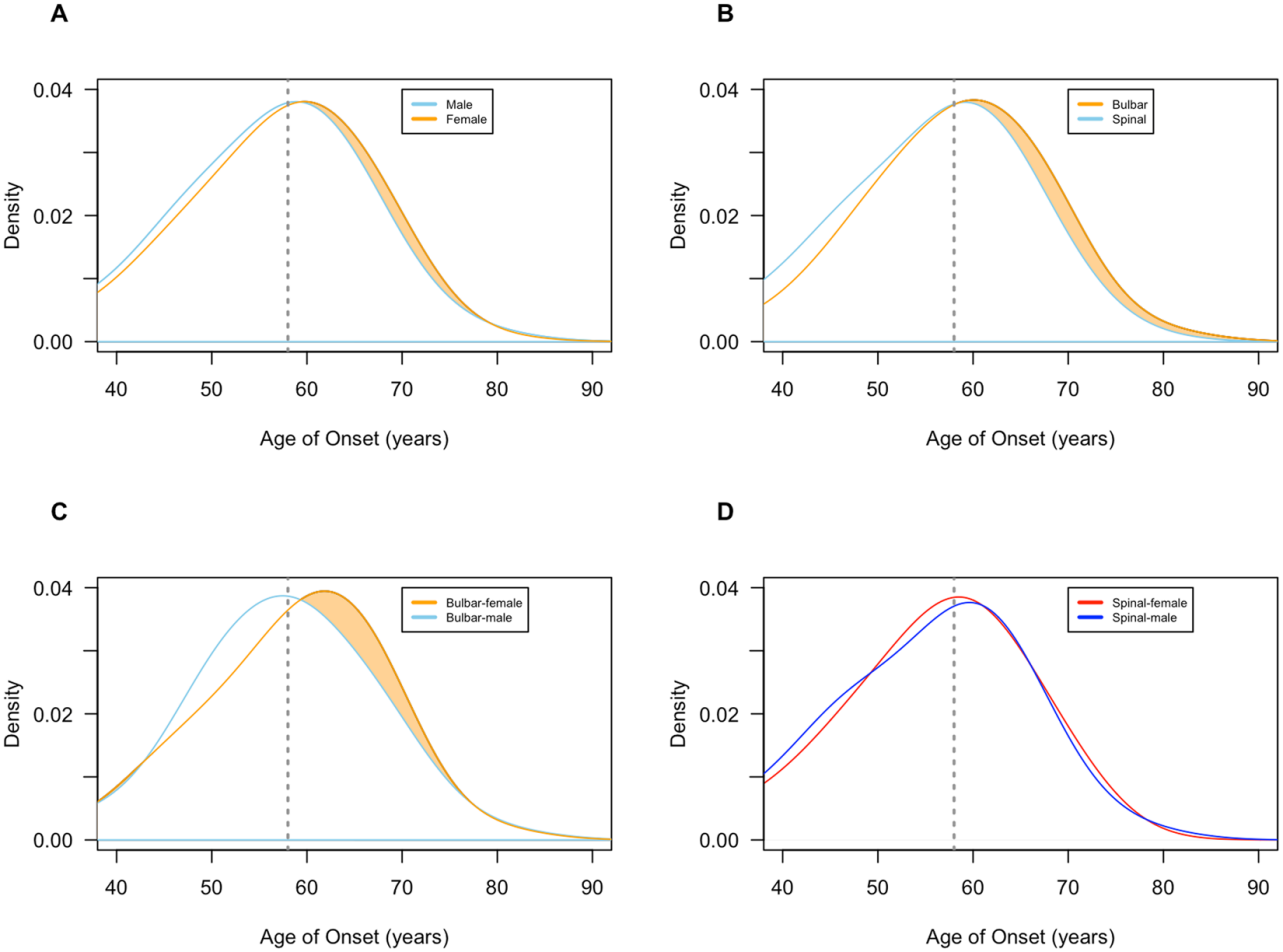
Density plots of age of onset among pathogenic *C9orf72* repeat expansion carriers. Density plot showing age of onset frequency distribution (**A**) among male (n = 393) and female (n = 357) ALS cases; (**B**) bulbar-onset (n = 256) and spinal-onset ALS cases (n = 481); (**C**) bulbar-onset male (n = 111) and bulbar-onset female (n = 111) ALS cases; (**D**) spinal-onset male (n = 273) and spinal-onset female (n = 208) cases. Shaded area represents differences between compared subgroups, and the dotted vertical line indicates median age of onset in the overall cohort (58.0 years of age).

Higher penetrance by age was also observed among cases with spinal-onset disease (median age of onset = 57.0 years of age, 95% CI: 56.0–58.0) compared to bulbar-onset (median age of onset = 59.0 years of age, 95% CI: 57.0–60.0; Log-rank test = 5.8, p-value = 0.02; see Fig. 3D). Intriguingly, density plots displaying the likelihood of developing disease at a given age indicated that this difference was driven by the older age of bulbar-onset disease among women compared to men (median age of disease onset among women = 60.0 years of age, 95% CI: 58.0–61.0; median age among men = 57.0, 95% CI: 56.0–59.0; see Fig. 4A–C). In contrast, there was no difference in age at onset of spinal-onset disease between genders (see Fig. 4D).

## Discussion

In this paper, we modeled the penetrance of the *C9orf72* hexanucleotide repeat expansion based on a large cohort drawn from published literature. We found that the penetrance of *C9orf72* repeat expansion exhibits incomplete, age-dependent penetrance, which confirms our previous findings and those of other groups^6, 9^. This more complete picture of *C9orf72* repeat expansion age-dependent penetrance will aid in the design and execution of clinical trials, and may be important – hopefully – in the future for decisions related to timing and administration of preventative therapy.

Our data show that family history of disease does not affect penetrance of *C9orf72*. This observation suggests that many sporadic cases are likely to be familial patients in disguise^10^. Genetic data showing that familial and sporadic cases of the *C9orf72* repeat expansion share the same haplotype also support this notion^6, 11, 12^. There are many reasons why familial disease might appear sporadic, including an incomplete family history and the death of previous generations before their disease development was evident. It is interesting to note that life expectancy in the United States did not reach 58, the median age at onset for *C9orf72* penetrance, until 1930^13^. This means that a member of a previous generation had an equal chance of dying from another disease, a phenomenen known as Gompertzian inter-disease competition^14^.

We also observed a slightly higher age-related penetrance among ALS compared to FTD cases. This phenomenon was mostly driven by individuals under the age of 58 years of age, whereas penetrance was identical above this age. The cause of this younger age of onset among ALS cases is not clear. One possibility is that the prodromal phase may be relatively short in ALS compared to FTD, reflecting the smaller baseline pool of motor neurons that must be destroyed to give rise to symptoms. It is also possible that the diagnosis of FTD is delayed as cognitive symptoms are more subtle than motor symptoms, or because of a more complex network of compensatory mechanisms may exist to delay overt symptoms. Alternatively, there may be an environmental, lifestyle, or hormonal factor that is driving the earlier onset of ALS cases, or that is acting in a protective manner among FTD cases^15, 16^.

The most pertinent finding regarding *C9orf72* penetrance was the older age of disease onset among female cases and among bulbar-onset cases. It has long been recognized that bulbar-onset ALS is more common among elderly females^17^, and these findings seem to point to a linkage between an individual’s sex and site of onset (see Fig. 1)^15, 18^. A hormonal or X-linked factor influencing disease type and the site of onset has been postulated, since women who develop ALS often have a later menarche and earlier menopause than controls^15, 19^.

We have examined the age-related penetrance associated with the *C9orf72* repeat expansion in a large cohort. It is important, however, to note the limitations of our approach. First, the relatively small number of cases (hundreds, rather than thousands) involved in our subgroup analysis limits power to detect real differences. Nevertheless, it is encouraging that the overall results from our current analysis are remarkably similar to what we previously reported^6, 20^. Second, an important question that remains is what is the true cumulative penetrance by the ninth decade of life. Our modeling indicates that disease penetrance associated with the *C9orf72* repeat expansion continues to rise beyond the seventh decade of life. For this reason, modeling of penetrance based on asymptomatic members within kindreds is likely to be inaccurate, as these individuals are younger and have not yet had an opportunity to develop disease and provide definitive information. Instead, analysis of a large cohort of elderly asymptomatic carriers will be required to definitively settle this issue.

A clinical trial of anti-sense oligonucleotide silencing the *C9orf72* repeat expansion is likely to commence enrollment soon^7^. If this trial is successful in slowing disease progression in patients, then an obvious evolution will be to determine if therapeutic intervention in asymptomatic *C9orf72* carriers can delay or even prevent disease onset. Similar prevention clinical trials are already underway in other neurodegenerative diseases (for example, Crenezumab in *PSEN1* mutation carriers, www.clinicaltrials.gov/ct2/show/NCT01998841). Exciting as this prospect may be, the variable age at onset among *C9orf72* repeat expansion carriers poses an obstacle to such trials, especially given the lack of a robust biomarker that can predict disease onset. Our data on *C9orf72* penetrance help to bridge this gap in our knowledge, and may be a critical element in the design of preventative clinical trials.

In conclusion, while familial and sporadic status does not influence age-related penetrance of *C9orf72*, penetrance by age does differ based on an individual’s sex, site of symptom onset, and the nature of the presenting neurological syndrome. These results will have important use for clinical research and genetic counseling for those with family history of disease. The next step is to determine the sources behind these variations, which may be genetic, environmental, or lifestyle factors.

## Materials and Methods

### Literature review

To gather the necessary raw data to calculate penetrance of the *C9orf72* mutation, we performed a literature search in PubMed using the term “C9orf72” combined with the MeSH terms “amyotrophic lateral sclerosis,” OR “motor neuron disease,” OR “dementia, frontotemporal”, OR “degeneration, frontotemporal lobar,” as well as specifying “humans.” The search was conducted in May 2016, and was limited to publications dating 2011 and later.

The literature search identified 71 articles reporting on a series of patients and neurologically normal individuals who were screened for presence of the *C9orf72* repeat expansion. Of these, 40 articles contained individual-level information on the age of symptom onset or age of screening among asymptomatic carriers. The entire list of publications can be found as Supplementary Information. The age and sex of those individuals with the repeat expansion was extracted from these publications, along with the country of origin, disease diagnosis, site of disease onset, and sporadic/familial status, if available.

### Cataloging of extracted data

The individual’s diagnosis could be categorized into one of nine options: ALS (n = 735), ALS-FTD (n = 76), FTD (n = 303), FTD-ALS (n = 20), Parkinson’s disease (n = 2), Alzheimer’s disease (n = 1), corticobasal syndrome (n = 1), olivopontocerebellar degeneration (n = 1), and neurologically normal (control subject, n = 23). As 96.9% of the patient diagnoses were either ALS or FTD, we focused on those two neurodegenerative diseases for our modeling of penetrance. The site of onset fell into one of four categories: bulbar, spinal, generalized, or cognitive. Disease pattern was classified as familial or sporadic based on the presence or absence of a family history of ALS or FTD. Data was available from 27 locations (see Supplemental Table S1 for full breakdown).

### Statistical analysis

To calculate age-related penetrance, we generated Kaplan-Meier plots in R statistical software (version 3.3.1) using the *Prodlim* (version 1.5.1) and *Survival* (version 2.38) packages^9, 21, 22^. Age at symptom onset was used as event time among affected individuals and age at screening and/or sample collection was used as censor time for asymptomatic carriers. Comparison between groups was performed using Log-rank tests within the *Survival* package. To model unaffected carriers that may not have been captured, we added 293 controls using the *sample* function in R to increase the portion of unaffected carriers in our cohort to 20% and randomly assigned them ages between 25 and 91 years. We used density estimation to generate posterior probabilities of developing symptoms based on age, gender, and site of symptom onset.

### Ethical approval

The analyses presented in this study were based on published literature, and the study was approved by the institutional review board of the National Institutes of Health (protocol number 03-AG-N329).

### Data availability

The complete data set used for this study, and the R programming code used to analyze these data are freely available at https://github.com/nam10/C9_Penetrance.

## Electronic supplementary material

Supplementary Info
