# Supplementary material for: Age-related penetrance of the C9orf72 repeat expansion
Source: Sci Rep. 2017 May 18;7:2116. doi: 10.1038/s41598-017-02364-1 (PMC5437033; doi:10.1038/s41598-017-02364-1)
Supplement: Supplementary file 1 — Supplementary Info [file 41598_2017_2364_MOESM1_ESM.pdf]

Supplementary Information

Age-related penetrance of the *C9orf72* repeat expansion

Natalie A. Murphy, Karissa C. Arthur, Pentti J. Tienari, Henry Houlden, Adriano Chiò & Bryan J. Traynor

Supplementary Table S1. Geographical origin of *C9orf72* repeat expansion carriers.

Area of Origin	Number of cases	Number of controls
Australia	45	1
Belgium	21	0
China	5	0
Denmark	3	0
Europe	2	0
Finland	149	2
France	76	1
Germany	41	2
Greece	16	0
Iran	5	0
Ireland	39	0
Israel	3	0
Italy	208	2
Japan	9	0
Netherlands	35	0
Portugal	3	0
Russia	9	0
Sardinia	22	0
Serbia	4	0
Singapore	1	0
Slovenia	5	0
Spain	5	0
Sweden	1	0
Taiwan	8	0
Turkey	29	0
United Kingdom	255	9
United States	149	6

Supplementary Table S2. Full data breakdown for *C9orf72* repeat expansion carriers. Table displaying the sample ID, gender, age of onset, diagnosis, site of onset, familial status, and geographical origin for the 1,170 individuals with the *C9orf72* repeat expansion included in the analysis.

Sample ID	Gender	Age of onset	Diagnosis	Site of onset	Familial status	Geographical origin	Reference
Abramycheva1	M	42	ALS	Spinal	Sporadic	Russia	1
Abramycheva2	M	53	ALS	Bulbar	Sporadic	Russia	1
Abramycheva3	M	63	ALS	Spinal	Sporadic	Russia	1
Abramycheva4	F	30	ALS	Bulbar	Sporadic	Russia	1
Abramycheva5	F	65	ALS	Spinal	Sporadic	Russia	1
Abramycheva6	F	65	ALS	Spinal	Familial	Russia	1
Abramycheva7	M	65	ALS	Spinal	Familial	Russia	1
Abramycheva8	M	45	ALS	Spinal	Familial	Russia	1
Abramycheva9	M	58	ALS	Bulbar	Sporadic	Russia	1
Al-Sarraj1	F	55	FTD	NA	Sporadic	UK	2
Al-Sarraj2	F	66	ALS	NA	Sporadic	UK	2
Al-Sarraj3	M	51	ALS	Bulbar	Familial	UK	2
Al-Sarraj4	M	50	ALS	Spinal	Sporadic	UK	2
Al-Sarraj5	M	69	ALS	Spinal	Sporadic	UK	2
Al-Sarraj6	F	65	FTD	NA	Sporadic	UK	2
Al-Sarraj7	F	35	ALS	Spinal	Sporadic	UK	2
Al-Sarraj8	M	51	FTD	NA	Sporadic	UK	2
Al-Sarraj9	M	61	ALS	Spinal	Sporadic	UK	2
Al-Sarraj10	F	42	ALS-FTD	Bulbar	Sporadic	UK	2
Al-Sarraj11	M	52	ALS	Spinal	Familial	UK	2
Al-Sarraj12	M	57	ALS	Spinal	Familial	UK	2
Al-Sarraj13	M	68	FTD	NA	Sporadic	UK	2
Al-Sarraj14	M	55	ALS	NA	Sporadic	UK	2
Alavi1	M	36	ALS	Spinal	Familial	Iran	3
Alavi2	F	34	ALS	Spinal	Sporadic	Iran	3
Alavi3	F	54	ALS	Spinal	Familial	Iran	3
Alavi4	M	57	ALS	Spinal	Familial	Iran	3
Alavi5	M	35	ALS	Spinal	Familial	Iran	3
Beck1	NA	54	Control	NA	NA	UK	4
Beck2	NA	54	Control	NA	NA	UK	4
Beck3	NA	54	Control	NA	NA	UK	4
Beck4	NA	54	Control	NA	NA	UK	4
Beck5	NA	54	Control	NA	NA	UK	4
Beck6	NA	54	Control	NA	NA	UK	4
Beck7	NA	54	Control	NA	NA	UK	4
Beck8	NA	54	Control	NA	NA	UK	4
Beck9	NA	54	Control	NA	NA	UK	4
Benussi1	F	58	FTD	NA	Familial	Italy	5
Benussi2	M	41	AD	NA	Familial	Italy	5
Benussi3	F	59	FTD	NA	Familial	Italy	5
Benussi4	F	46	FTD	NA	Familial	Italy	5

Benussi5	M	62	FTD	NA	Familial	Italy	5
Benussi6	F	58	FTD	NA	Familial	Italy	5
Benussi7	F	49	FTD	NA	Familial	Italy	5
Benussi8	M	47	FTD	NA	Familial	Italy	5
Benussi9	M	56	FTD	NA	Familial	Italy	5
Benussi10	M	61	FTD	NA	Familial	Italy	5
Benussi11	M	55	FTD	NA	Familial	Italy	5
Benussi12	M	52	FTD	NA	Familial	Italy	5
Benussi13	M	58	FTD	NA	Familial	Italy	5
Benussi14	F	57	FTD	NA	Familial	Italy	5
Benussi15	M	80	FTD	NA	Familial	Italy	5
Benussi16	F	47	FTD	NA	Sporadic	Italy	5
Benussi17	F	70	FTD	NA	Sporadic	Italy	5
Benussi18	M	72	FTD-ALS	NA	Sporadic	Italy	5
Benussi19	M	56	FTD	NA	Sporadic	Italy	5
Benussi20	F	57	FTD	NA	Sporadic	Italy	5
Benussi21	M	57	FTD-ALS	NA	Sporadic	Italy	5
Benussi22	F	51	FTD	NA	Sporadic	Italy	5
Benussi23	F	45	FTD	NA	Sporadic	Italy	5
Benussi24	M	49	FTD-ALS	NA	Sporadic	Italy	5
Benussi25	M	59	FTD	NA	Sporadic	Italy	5
Benussi26	F	62	FTD	NA	Sporadic	Italy	5
Benussi27	M	58	FTD	NA	Sporadic	Italy	5
Benussi28	M	56	FTD-ALS	NA	Sporadic	Italy	5
Benussi29	F	47	ALS	NA	Sporadic	Italy	5
Cerami1	M	54	FTD	NA	NA	Italy	6
Cerami2	F	68	FTD	NA	NA	Italy	6
Cerami3	F	68	FTD	NA	NA	Italy	6
Cerami4	M	61	FTD	NA	NA	Italy	6
Chester1	F	40	ALS	Bulbar	Familial	Portugal	7
Chester2	M	40	ALS	Bulbar	Familial	Portugal	7
Chester3	F	47	ALS	Bulbar	Familial	Portugal	7
Chio1	F	64	ALS	Spinal	Familial	Italy	8
Chio2	M	33	ALS	Spinal	Familial	Italy	8
Chio3	M	63	ALS	Spinal	Familial	Italy	8
Chio4	F	62	ALS	Spinal	Familial	Italy	8
Chio5	F	62	ALS	Bulbar	Familial	Italy	8
Chio6	F	36	ALS-FTD	Spinal	Familial	Italy	8
Chio7	M	45	ALS	Spinal	Familial	Italy	8
Chio8	M	59	ALS	Spinal	Familial	Italy	8
Chio9	F	78	FTD	NA	Familial	Italy	8
Chio10	M	59	FTD	NA	Familial	Italy	8
Chio11	F	59	ALS-FTD	Bulbar	Familial	Italy	8
Chio12	M	62	FTD	NA	Familial	Italy	8
Cooper-Knock1	M	69	ALS	Spinal	Familial	UK	9
Cooper-Knock2	F	59	ALS	Spinal	Familial	UK	9
Cooper-Knock3	M	42	ALS	Spinal	Familial	UK	9

Cooper-Knock4	M	66	ALS	Bulbar	Familial	UK	9
Cooper-Knock5	M	64	ALS	Spinal	Familial	UK	9
Cooper-Knock6	F	62	ALS	Bulbar	Familial	UK	9
Cooper-Knock7	F	56	ALS	Spinal	Familial	UK	9
Cooper-Knock8	F	50	ALS	Bulbar	Familial	UK	9
Cooper-Knock9	M	47	ALS	General	Familial	UK	9
Cooper-Knock10	F	63	ALS	Cognitive	Familial	UK	9
Cooper-Knock11	F	61	ALS	Spinal	Familial	UK	9
Cooper-Knock12	F	65	ALS	Bulbar	Sporadic	UK	9
Cooper-Knock13	F	67	NA	Spinal	Sporadic	UK	9
Cooper-Knock14	M	63	ALS	Spinal	Sporadic	UK	9
Cooper-Knock15	M	56	ALS	Bulbar	Sporadic	UK	9
Cooper-Knock16	F	61	ALS	Bulbar	Sporadic	UK	9
Cooper-Knock17	F	58	ALS	Spinal	Sporadic	UK	9
Cooper-Knock18	F	61	ALS	Spinal	Sporadic	UK	9
Cooper-Knock19	M	62	ALS	Bulbar	Sporadic	UK	9
Cooper-Knock20	M	45	ALS	Spinal	Sporadic	UK	9
Cooper-Knock21	F	51	ALS	General	Sporadic	UK	9
Cooper-Knock22	F	47	ALS	Spinal	Familial	UK	9
Cooper-Knock23	M	61	ALS	Spinal	Familial	UK	9
Cooper-Knock24	F	27	ALS	Bulbar	Familial	UK	9
Cooper-Knock25	F	50	ALS	Bulbar	Familial	UK	9
Cooper-Knock26	F	48	ALS	Spinal	Familial	UK	9
Cooper-Knock27	M	48	ALS	Bulbar	Familial	UK	9
Cooper-Knock28	F	61	ALS	Spinal	Familial	UK	9
Cooper-Knock29	F	44	ALS	Bulbar	Familial	UK	9
Cooper-Knock30	F	61	NA	Bulbar	Familial	UK	9
Cooper-Knock31	F	64	ALS	Spinal	Familial	UK	9
Cooper-Knock32	F	45	ALS	Bulbar	Familial	UK	9
Cooper-Knock33	M	51	ALS	Spinal	Familial	UK	9
Cooper-Knock34	M	65	ALS	Spinal	Familial	UK	9
Cooper-Knock35	M	63	ALS	General	Familial	UK	9
Cooper-Knock36	F	37	ALS	Spinal	Familial	UK	9
Cooper-Knock37	M	56	ALS	Spinal	Sporadic	UK	9
Cooper-Knock38	M	56	ALS	Spinal	Sporadic	UK	9
Cooper-Knock39	F	66	ALS	Bulbar	Sporadic	UK	9
Cooper-Knock40	M	60	ALS	Bulbar	Sporadic	UK	9
Cooper-Knock41	F	50	ALS	Spinal	Sporadic	UK	9
Cooper-Knock42	M	60	ALS	Spinal	Sporadic	UK	9
Cooper-Knock43	M	43	ALS	Spinal	Sporadic	UK	9
Cooper-Knock44	M	64	ALS	Spinal	Sporadic	UK	9
Cooper-Knock45	M	74	ALS	Spinal	Sporadic	UK	9
Cooper-Knock46	M	58	ALS	Spinal	Sporadic	UK	9
Cooper-Knock47	F	57	ALS	Spinal	Sporadic	UK	9
Cooper-Knock48	F	62	ALS	Spinal	Sporadic	UK	9
Cooper-Knock49	F	57	ALS	General	Sporadic	UK	9
Cooper-Knock50	M	60	ALS	Bulbar	Sporadic	UK	9

Cooper-Knock51	M	63	ALS	Spinal	Sporadic	UK	9
Cooper-Knock52	M	61	ALS	Spinal	Sporadic	UK	9
Cooper-Knock53	M	51	ALS	Bulbar	Sporadic	UK	9
Cooper-Knock54	M	50	ALS	Bulbar	Sporadic	UK	9
Cooper-Knock55	F	59	ALS	Spinal	Sporadic	UK	9
Cooper-Knock56	F	71	ALS	Spinal	Sporadic	UK	9
Cooper-Knock57	F	65	ALS	Spinal	Sporadic	UK	9
Cooper-Knock58	F	52	ALS	Spinal	Sporadic	UK	9
Cooper-Knock59	F	63	ALS	Bulbar	Sporadic	UK	9
Cooper-Knock60	F	71	ALS	Spinal	Sporadic	UK	9
Cooper-Knock61	M	65	ALS	Spinal	Sporadic	UK	9
Dobson-Stone1	F	53	FTD	Cognitive	NA	Australia	10
Dobson-Stone2	M	60	FTD-ALS	NA	NA	Australia	10
Dobson-Stone3	F	62	FTD	Cognitive	NA	Australia	10
Dobson-Stone4	M	45	FTD	Cognitive	NA	Australia	10
Dobson-Stone5	M	49	FTD	Cognitive	NA	Australia	10
Dobson-Stone6	M	65	FTD-ALS	NA	NA	Australia	10
Dobson-Stone7	M	48	FTD	Cognitive	NA	Australia	10
Dobson-Stone8	F	61	FTD	Cognitive	NA	Australia	10
Dobson-Stone9	F	47	FTD-ALS	NA	NA	Australia	10
Dobson-Stone10	M	69	AD	NA	NA	Australia	10
Dobson-Stone11	M	63	FTD	Cognitive	NA	Australia	10
Dobson-Stone12	M	55	FTD	Cognitive	NA	Australia	10
Dobson-Stone13	M	60	AD	NA	NA	Australia	10
Dobson-Stone14	M	64	FTD	Cognitive	NA	Australia	10
Dobson-Stone15	F	60	FTD-ALS	NA	NA	Australia	10
Dobson-Stone16	M	56	FTD	Cognitive	NA	Australia	10
Dobson-Stone17	M	60	AD	NA	NA	Australia	10
Dobson-Stone18	F	89	Control	NA	NA	Australia	10
Dobson-Stone19	M	52	ALS-FTD	NA	Familial	Australia	11
Dobson-Stone20	M	43	FTD	Cognitive	Familial	Australia	11
Dobson-Stone21	M	57	FTD-ALS	Cognitive	Familial	Australia	11
Dobson-Stone22	M	59	FTD-ALS	Cognitive	Familial	Australia	11
Dobson-Stone23	F	52	FTD-ALS	NA	Familial	Australia	11
Dobson-Stone24	M	65	FTD-ALS	NA	Familial	Australia	11
Dobson-Stone25	M	41	AD	NA	Sporadic	Australia	11
Dobson-Stone26	M	55	FTD	NA	Familial	Australia	11
Dobson-Stone27	F	62	ALS-FTD	NA	Familial	Australia	11
Dobson-Stone28	F	64	FTD	NA	Familial	Australia	11
Dobson-Stone29	M	58	ALS-FTD	NA	Familial	Australia	11
Dobson-Stone30	F	41	ALS-FTD	NA	Familial	Australia	11
Dobson-Stone31	F	56	FTD	Cognitive	Familial	Spain	11
Dobson-Stone32	M	64	FTD	Cognitive	Sporadic	Spain	11
Dobson-Stone33	F	63	FTD	Cognitive	Familial	Spain	11
Dobson-Stone34	F	70	FTD	NA	Sporadic	Spain	11
Dobson-Stone35	M	51	FTD	Cognitive	Familial	Spain	11
Dombroski1	M	53	ALS	NA	Sporadic	US	12

Ferrari1	M	53	FTD	Cognitive	Familial	US	13
Ferrari2	M	60	ALS-FTD	Spinal	Sporadic	US	13
Ferrari3	M	61	ALS-FTD	Bulbar	Familial	US	13
Ferrari4	M	59	FTD	Cognitive	Familial	US	13
ND07551	M	66	Control	NA	NA	US	13
Fratta1	NA	57	ALS	Bulbar	Sporadic	UK	14
Galimberti1	M	81	Control	NA	NA	Italy	15
Galimberti2	F	80	Control	NA	NA	Italy	15
Galimberti3	F	57	FTD	Cognitive	Familial	Italy	16
Galimberti4	F	75	FTD	Cognitive	Sporadic	Italy	16
Galimberti5	M	52	FTD	Cognitive	Familial	Italy	16
Galimberti6	F	70	FTD	Cognitive	Sporadic	Italy	16
Galimberti7	F	74	FTD	Cognitive	Sporadic	Italy	16
Galimberti8	M	57	FTD-ALS	Cognitive	Familial	Italy	16
Galimberti9	F	68	FTD	NA	Sporadic	Italy	16
Galimberti10	F	72	FTD	Cognitive	Familial	Italy	16
Galimberti11	M	59	FTD	Cognitive	Sporadic	Italy	16
Galimberti12	M	49	FTD	Cognitive	Familial	Italy	16
Galimberti13	F	73	FTD-ALS	Cognitive	Familial	Italy	16
Galimberti14	F	77	FTD	Cognitive	Sporadic	Italy	16
Galimberti15	M	58	FTD	Cognitive	Familial	Italy	16
Galimberti16	F	66	FTD	Cognitive	Familial	Italy	16
Galimberti17	F	60	FTD	Cognitive	Familial	Italy	16
Galimberti18	M	62	FTD	NA	Sporadic	Italy	16
Galimberti19	F	71	FTD	Cognitive	Familial	Italy	16
Galimberti20	M	65	FTD	Cognitive	Familial	Italy	16
Galimberti21	M	66	FTD-ALS	Cognitive	Sporadic	Italy	16
Galimberti22	F	60	FTD	Cognitive	Sporadic	Italy	16
Galimberti23	M	48	FTD-ALS	Cognitive	Familial	Italy	16
Galimberti24	F	49	FTD	Cognitive	Sporadic	Italy	16
Galimberti25	F	65	FTD	Cognitive	Sporadic	Italy	16
Galimberti26	M	NA	FTD	Cognitive	Sporadic	Italy	16
Galimberti27	F	50	FTD	Cognitive	Familial	Italy	16
Galimberti28	F	55	FTD	Cognitive	Familial	Italy	16
Galimberti29	M	70	FTD	Cognitive	Familial	Italy	16
Galimberti30	F	74	FTD	Cognitive	Familial	Italy	16
Galimberti31	F	60	FTD	Cognitive	Familial	Italy	16
Galimberti32	M	71	FTD	Cognitive	Familial	Italy	16
Galimberti33	M	52	FTD-ALS	Cognitive	Familial	Italy	16
Galimberti34	F	71	FTD	Cognitive	Familial	Italy	16
Galimberti35	F	65	FTD-ALS	Cognitive	Familial	Italy	16
Galimberti36	M	71	FTD-ALS	Cognitive	Sporadic	Italy	16
Galimberti37	M	64	FTD	Cognitive	Familial	Italy	16
Galimberti38	F	76	FTD	Cognitive	Sporadic	Italy	16
Galimberti39	M	66	FTD	Cognitive	Familial	Italy	16
Galimberti40	F	57	FTD	Cognitive	Familial	Italy	16
Galimberti41	F	62	FTD-ALS	Cognitive	Sporadic	Italy	16

Gijselinck1	M	57	FTD	Cognitive	Familial	Belgium	17
Gijselinck2	F	51	FTD	Cognitive	Familial	Belgium	17
Gijselinck3	M	53	FTD	Cognitive	Familial	Belgium	17
Gijselinck4	F	42	FTD	Cognitive	Sporadic	Belgium	17
Gijselinck5	M	69	FTD	Cognitive	Familial	Belgium	17
Gijselinck6	F	45	FTD	Cognitive	Familial	Belgium	17
Gijselinck7	M	46	FTD	Cognitive	Familial	Belgium	17
Gijselinck8	M	53	FTD	Cognitive	Familial	Belgium	17
Gijselinck9	M	58	FTD	Cognitive	Sporadic	Belgium	17
Gijselinck10	M	49	FTD	Cognitive	Sporadic	Belgium	17
Gijselinck11	F	65	ALS-FTD	Bulbar	Familial	Belgium	17
Gijselinck12	F	60	ALS-FTD	Bulbar	Familial	Belgium	17
Gijselinck13	F	69	ALS-FTD	Spinal	Familial	Belgium	17
Gijselinck14	F	65	ALS-FTD	Bulbar	Familial	Belgium	17
Gijselinck15	M	50	ALS-FTD	Bulbar	Familial	Belgium	17
Gijselinck16	M	60	ALS	Bulbar	Familial	Belgium	17
Gijselinck17	F	47	ALS	Spinal	Familial	Belgium	17
Gijselinck18	M	55	ALS	Spinal	Familial	Belgium	17
Gijselinck19	F	62	ALS	Spinal	Sporadic	Belgium	17
Gijselinck20	M	38	ALS	Spinal	Sporadic	Belgium	17
Gijselinck21	M	65	ALS	Spinal	NA	Belgium	17
Harms13	NA	73	Control	NA	NA	US	18
Harms1	M	53	ALS	Spinal	Sporadic	US	19
Harms2	M	46	ALS	Spinal	Familial	US	19
Harms3	M	72	ALS-FTD	Bulbar	Familial	US	19
Harms4	F	52	ALS	Spinal	Familial	US	19
Harms5	M	49	ALS	Bulbar	Sporadic	US	19
Harms6	F	70	ALS	Spinal	Sporadic	US	19
Harms7	M	68	ALS	Spinal	Sporadic	US	19
Harms8	F	43	ALS	Spinal	Sporadic	US	19
Harms9	F	62	ALS-FTD	Spinal	Familial	US	19
Harms10	NA	50	Control	NA	NA	US	20
Harms11	NA	67	Control	NA	NA	US	20
Harms12	NA	71	Control	NA	NA	US	20
He1	M	58	ALS	Spinal	Sporadic	China	21
He2	NA	58	ALS	Spinal	Sporadic	China	21
He3	M	54	ALS	Bulbar	Sporadic	China	21
Ishiura1	F	72	ALS	Bulbar	Sporadic	Japan	22
Ishiura2	F	71	ALS	Spinal	Familial	Japan	22
Ishiura3	F	41	ALS	Spinal	Sporadic	Japan	22
Jiao1	M	47	ALS-FTD	Spinal	Familial	China	23
Jiao2	M	67	FTD	Cognitive	Sporadic	China	23
Kaivorinne1	F	58	FTD	NA	Sporadic	Finland	24
Kaivorinne2	M	57	FTD	NA	Familial	Finland	24
Kaivorinne3	F	64	FTD	NA	Familial	Finland	24
Kaivorinne4	F	60	FTD	NA	Familial	Finland	24
Kaivorinne5	M	54	FTD	NA	Familial	Finland	24

Kaivorinne6	F	47	FTD	NA	Familial	Finland	24
Kaivorinne7	F	51	FTD	NA	Sporadic	Finland	24
Kaivorinne8	M	55	FTD	NA	Familial	Finland	24
Kaivorinne9	M	54	FTD	NA	Familial	Finland	24
Kaivorinne10	M	46	FTD	NA	Familial	Finland	24
Kaivorinne11	F	59	FTD	NA	Familial	Finland	24
Kaivorinne12	M	64	FTD	NA	Familial	Finland	24
Kaivorinne13	F	60	FTD	NA	Sporadic	Finland	24
Kaivorinne14	M	51	FTD	NA	Sporadic	Finland	24
Kaivorinne15	M	64	FTD	NA	Sporadic	Finland	24
Kaivorinne16	F	63	FTD	NA	Familial	Finland	24
Kaivorinne17	F	59	ALS-FTD	Bulbar	Sporadic	Finland	24
Kaivorinne18	F	54	ALS-FTD	Bulbar	Familial	Finland	24
Kaivorinne19	M	70	ALS-FTD	Bulbar	Sporadic	Finland	24
Kaivorinne20	M	57	ALS-FTD	Bulbar	Sporadic	Finland	24
Kaivorinne21	F	60	ALS-FTD	Spinal	Sporadic	Finland	24
Kaivorinne22	F	66	ALS-FTD	Bulbar	Sporadic	Finland	24
Kandiah1	F	42	FTD	Cognitive	Familial	Singapore	25
Kenna1	M	35	ALS	Spinal	Familial	Ireland	26
Kenna2	M	34	ALS	General	Sporadic	Ireland	26
Kenna3	F	51	ALS	Spinal	Sporadic	Ireland	26
Kenna4	F	60	ALS	Bulbar	Sporadic	Ireland	26
Kenna5	M	47	ALS	Bulbar	Sporadic	Ireland	26
Kenna6	M	66	ALS	Spinal	Sporadic	Ireland	26
Kenna7	M	44	ALS	Spinal	Sporadic	Ireland	26
Kenna8	F	43	ALS	Bulbar	Sporadic	Ireland	26
Kenna9	F	52	ALS	Bulbar	Sporadic	Ireland	26
Kenna10	M	68	ALS	Spinal	Sporadic	Ireland	26
Kenna11	F	82	ALS	Bulbar	Familial	Ireland	26
Kenna12	F	42	ALS	Spinal	Sporadic	Ireland	26
Kenna13	F	55	ALS	Spinal	Sporadic	Ireland	26
Kenna14	F	44	ALS	General	Familial	Ireland	26
Kenna15	M	55	ALS	Spinal	Sporadic	Ireland	26
Kenna16	M	62	ALS-FTD	Spinal	Sporadic	Ireland	26
Kenna17	M	56	ALS-FTD	Spinal	Familial	Ireland	26
Kenna18	F	44	ALS	Spinal	Sporadic	Ireland	26
Kenna19	F	65	ALS-FTD	Spinal	Familial	Ireland	26
Kenna20	M	65	ALS-FTD	Spinal	Sporadic	Ireland	26
Kenna21	M	48	ALS	Spinal	Sporadic	Ireland	26
Kenna22	F	63	ALS	Bulbar	Sporadic	Ireland	26
Kenna23	F	49	ALS	Spinal	Sporadic	Ireland	26
Kenna24	F	62	ALS	Spinal	Sporadic	Ireland	26
Kenna25	M	59	ALS	Bulbar	Familial	Ireland	26
Kenna26	F	64	ALS	Bulbar	Familial	Ireland	26
Kenna27	F	65	ALS	Bulbar	Familial	Ireland	26
Kenna28	M	66	ALS	Bulbar	Familial	Ireland	26
Kenna29	M	36	ALS-FTD	Spinal	Sporadic	Ireland	26

Kenna30	M	59	ALS	Bulbar	Sporadic	Ireland	26
Kenna31	F	72	ALS	Spinal	Sporadic	Ireland	26
Kenna32	M	44	ALS	Bulbar	Sporadic	Ireland	26
Kenna33	M	59	ALS	Spinal	Familial	Ireland	26
Kenna34	F	52	ALS	Spinal	Familial	Ireland	26
Kenna35	M	55	ALS	Spinal	Sporadic	Ireland	26
Kenna36	M	58	ALS-FTD	Bulbar	Familial	Ireland	26
Kenna37	M	65	ALS	Spinal	Familial	Ireland	26
Kenna38	M	61	ALS	Spinal	Sporadic	Ireland	26
Konno1	M	61	ALS	Spinal	Sporadic	Japan	27
Konno2	F	63	ALS	Bulbar	Sporadic	Japan	27
LeBer1	M	55	PD	Spinal	Familial	France	28
LeBer2	F	48	PD	Spinal	Sporadic	France	28
LeBer3	F	30	ALS	Spinal	Familial	France	28
LeBer4	M	45	FTD	Cognitive	Sporadic	France	28
Lindquist1	M	42	CBS	NA	Familial	Denmark	29
Lindquist2	F	53	OPCD	NA	Familial	Denmark	29
Lindquist3	M	62	ALS-FTD	Bulbar	Sporadic	Denmark	29
Majounie1	M	65	AD	NA	Familial	Europe	30
Majounie2	M	71	AD	NA	Familial	Europe	30
AUS2337	F	55	ALS	Bulbar	Sporadic	Australia	31
AUS1039	F	58	ALS	Spinal	Sporadic	Australia	31
AUS2312	F	58	ALS	Spinal	Sporadic	Australia	31
AUS2579	F	59	ALS	Bulbar	Sporadic	Australia	31
AUS2423	F	60	ALS	Bulbar	Sporadic	Australia	31
AUS1090	F	60	ALS	Spinal	Sporadic	Australia	31
AUS2359	F	64	ALS	Bulbar	Sporadic	Australia	31
AUS2516	F	67	ALS	Bulbar	Sporadic	Australia	31
AUS2193	F	70	ALS	Bulbar	Sporadic	Australia	31
AUS0318	M	50	ALS	Spinal	Sporadic	Australia	31
AUS2721	M	54	ALS	Spinal	Sporadic	Australia	31
AUS0253	M	55	ALS	Spinal	Sporadic	Australia	31
AUS2748	M	57	ALS	Spinal	Sporadic	Australia	31
AUS2101	M	67	ALS	Bulbar	Sporadic	Australia	31
M430	F	42	ALS	Bulbar	Sporadic	Finland	31
M461	F	42	ALS	NA	Familial	Finland	31
M375	F	43	ALS	NA	Familial	Finland	31
M69	F	43	ALS	Spinal	Sporadic	Finland	31
M463	F	44	ALS	NA	Sporadic	Finland	31
M427	F	47	ALS	Spinal	Familial	Finland	31
M36	F	47	ALS	Spinal	Sporadic	Finland	31
M355	F	48	ALS	Bulbar	Familial	Finland	31
M45	F	48	ALS	Spinal	Sporadic	Finland	31
M40	F	49	ALS	Bulbar	Sporadic	Finland	31
M234	F	49	ALS	NA	Sporadic	Finland	31
M113	F	50	ALS	Bulbar	Familial	Finland	31
M228	F	51	ALS	Bulbar	Sporadic	Finland	31

M472	F	52	ALS	Bulbar	Familial	Finland	31
M88	F	52	ALS	Spinal	Familial	Finland	31
M71	F	53	ALS	Spinal	Familial	Finland	31
M217	F	53	ALS	Spinal	Familial	Finland	31
M11	F	53	ALS	Spinal	Sporadic	Finland	31
M46	F	53	ALS	Spinal	Sporadic	Finland	31
M147	F	55	ALS	Bulbar	Familial	Finland	31
M344	F	55	ALS	Bulbar	Familial	Finland	31
M341	F	55	ALS	NA	Familial	Finland	31
M51	F	55	ALS	Spinal	Familial	Finland	31
M242	F	55	ALS	Spinal	Familial	Finland	31
M218	F	55	ALS	Spinal	Sporadic	Finland	31
M350	F	56	ALS	Spinal	Sporadic	Finland	31
M326	F	57	ALS	Spinal	Familial	Finland	31
M399	F	57	ALS	Spinal	Familial	Finland	31
M114	F	57	ALS	Spinal	Sporadic	Finland	31
M396	F	57	ALS	Spinal	Sporadic	Finland	31
M23	F	58	ALS	Bulbar	Sporadic	Finland	31
M102	F	58	ALS	Bulbar	Sporadic	Finland	31
M16	F	58	ALS	Spinal	Familial	Finland	31
M248	F	58	ALS	Spinal	Sporadic	Finland	31
M415	F	59	ALS	Spinal	Sporadic	Finland	31
M101	F	60	ALS	Bulbar	Familial	Finland	31
M269	F	60	ALS	Spinal	Sporadic	Finland	31
M167	F	61	ALS	Bulbar	Familial	Finland	31
M343	F	61	ALS	Bulbar	Sporadic	Finland	31
M469	F	61	ALS	NA	Familial	Finland	31
M245	F	62	ALS	Bulbar	Sporadic	Finland	31
M354	F	62	ALS	NA	Familial	Finland	31
M60	F	63	ALS	Spinal	Sporadic	Finland	31
M150	F	64	ALS	Spinal	Sporadic	Finland	31
M137	F	65	ALS	Bulbar	Familial	Finland	31
M369	F	65	ALS	Bulbar	Sporadic	Finland	31
M158	F	65	ALS	Spinal	Sporadic	Finland	31
M257	F	65	ALS	Spinal	Sporadic	Finland	31
M73	F	66	ALS	Spinal	Familial	Finland	31
M170	F	67	ALS	Bulbar	Sporadic	Finland	31
M72	F	67	ALS	Spinal	Sporadic	Finland	31
M262	F	68	ALS	Bulbar	Familial	Finland	31
M392	F	68	ALS	Spinal	Familial	Finland	31
M24	F	71	ALS	Bulbar	Sporadic	Finland	31
M272	M	35	ALS	Spinal	Sporadic	Finland	31
M202	M	37	ALS	Bulbar	Sporadic	Finland	31
M230	M	37	ALS	Spinal	Sporadic	Finland	31
M387	M	38	ALS	NA	Sporadic	Finland	31
M370	M	39	ALS	NA	Sporadic	Finland	31
M169	M	41	ALS	Spinal	Sporadic	Finland	31

M312	M	43	ALS	Spinal	Familial	Finland	31
M121	M	44	ALS	Spinal	Familial	Finland	31
M332	M	46	ALS	Spinal	Familial	Finland	31
M327	M	46	ALS	Spinal	Sporadic	Finland	31
M56	M	47	ALS	Bulbar	Familial	Finland	31
M83	M	47	ALS	Spinal	Sporadic	Finland	31
M324	M	47	ALS	Spinal	Sporadic	Finland	31
M42	M	48	ALS	Bulbar	Familial	Finland	31
M237	M	48	ALS	Spinal	Familial	Finland	31
M75	M	50	ALS	Spinal	Familial	Finland	31
M115	M	52	ALS	Bulbar	Sporadic	Finland	31
M220	M	52	ALS	Bulbar	Sporadic	Finland	31
M303	M	52	ALS	Spinal	Sporadic	Finland	31
M316	M	54	ALS	Spinal	Familial	Finland	31
M50	M	55	ALS	Spinal	Familial	Finland	31
M58	M	55	ALS	Spinal	Sporadic	Finland	31
M39	M	56	ALS	Bulbar	Familial	Finland	31
M493	M	56	ALS	Bulbar	Sporadic	Finland	31
M141	M	56	ALS	Spinal	Familial	Finland	31
M349	M	56	ALS	Spinal	Familial	Finland	31
M340	M	57	ALS	Bulbar	Sporadic	Finland	31
M458	M	57	ALS	Bulbar	Sporadic	Finland	31
M172	M	57	ALS	Spinal	Familial	Finland	31
M335	M	58	ALS	Spinal	Familial	Finland	31
M93	M	59	ALS	Bulbar	Familial	Finland	31
M14	M	59	ALS	Bulbar	Sporadic	Finland	31
M66	M	59	ALS	Spinal	Familial	Finland	31
M63	M	59	ALS	Spinal	Sporadic	Finland	31
M96	M	59	ALS	Spinal	Sporadic	Finland	31
M222	M	59	ALS	Spinal	Sporadic	Finland	31
M301	M	61	ALS	Spinal	Sporadic	Finland	31
M52	M	62	ALS	Spinal	Familial	Finland	31
M97	M	62	ALS	Spinal	Familial	Finland	31
M373	M	63	ALS	Spinal	Sporadic	Finland	31
M84	M	64	ALS	Bulbar	Sporadic	Finland	31
M444	M	64	ALS	Spinal	Sporadic	Finland	31
M28	M	65	ALS	Spinal	Familial	Finland	31
M280	M	65	ALS	Spinal	Familial	Finland	31
M338	M	65	ALS	Spinal	Familial	Finland	31
M53	M	65	ALS	Spinal	Sporadic	Finland	31
M92	M	66	ALS	Bulbar	Sporadic	Finland	31
M94	M	66	ALS	Spinal	Sporadic	Finland	31
M13	M	69	ALS	Bulbar	Sporadic	Finland	31
M76	M	71	ALS	Bulbar	Familial	Finland	31
M250	M	77	ALS	Spinal	Familial	Finland	31
M306	F	47	FTD	Cognitive	Familial	Finland	31
FTD330	F	51	FTD	Cognitive	Sporadic	Finland	31

M51	F	54	FTD	Cognitive	Familial	Finland	31
FTD212	F	58	FTD	Cognitive	Sporadic	Finland	31
M352	F	59	FTD	Cognitive	Familial	Finland	31
FTD047	F	59	FTD	Cognitive	Sporadic	Finland	31
M240	F	60	FTD	Cognitive	Familial	Finland	31
FTD200	F	60	FTD	Cognitive	Sporadic	Finland	31
FTD014	F	60	FTD	Cognitive	Sporadic	Finland	31
M109	F	63	FTD	Cognitive	Familial	Finland	31
M225	F	64	FTD	Cognitive	Familial	Finland	31
FTD328	F	66	FTD	Cognitive	Sporadic	Finland	31
M348	M	46	FTD	Cognitive	Familial	Finland	31
FTD063	M	51	FTD	Cognitive	Sporadic	Finland	31
M257	M	54	FTD	Cognitive	Familial	Finland	31
M337	M	54	FTD	Cognitive	Familial	Finland	31
M336	M	55	FTD	Cognitive	Familial	Finland	31
M222	M	57	FTD	Cognitive	Familial	Finland	31
FTD191	M	57	FTD	Cognitive	Sporadic	Finland	31
M353	M	64	FTD	Cognitive	Familial	Finland	31
FTD091	M	64	FTD	Cognitive	Sporadic	Finland	31
M66	M	70	FTD	Cognitive	Familial	Finland	31
VAN537	F	91	Control	NA	NA	Finland	31
VAN301	M	86	Control	NA	NA	Finland	31
DFT-NIM-SAN-133	F	48	FTD	Cognitive	Familial	France	31
DFT-TEN-CAR-233	F	57	FTD	NA	Familial	France	31
DFT-CLE-RAV-536	F	60	FTD	Cognitive	Familial	France	31
DFT-SAL-BRE-257	F	60	FTD	NA	Sporadic	France	31
DFT-NAN-CAR-092	F	61	FTD	NA	Sporadic	France	31
DFT-SAL-GRA-250	F	62	FTD	Cognitive	Sporadic	France	31
DFT-LIM-VER-706	F	64	FTD	NA	Sporadic	France	31
DFT-NAN-BRO-102	F	67	FTD	NA	Familial	France	31
DFT-SAL-JOL-172	F	68	FTD	Cognitive	Familial	France	31
DFT-BRI-DEL-221	F	68	FTD	NA	Familial	France	31
DFT-TOU-BOU-209	F	68	FTD	NA	Familial	France	31
DFT-REN-LOR-030	F	69	FTD	NA	Familial	France	31
DFT-BRI-SAI-196	F	75	FTD	NA	Familial	France	31
DFT-GEN-RHO-198	M	30	FTD	NA	Familial	France	31
DFT-BOR-BAU-126	M	35	FTD	NA	Familial	France	31
DFT-SAL-GAR-014	M	42	FTD	Cognitive	Sporadic	France	31
DFT-CAE-LEL-151	M	47	FTD	NA	Familial	France	31
DFT-SAI-LAC-029	M	50	FTD	Cognitive	Sporadic	France	31
DFT-REN-TAR-006	M	50	FTD	NA	Familial	France	31
DFT-TEN-ROU-173	M	50	FTD	NA	Familial	France	31
DFT-NAN-ETI-455	M	51	FTD	NA	Sporadic	France	31
DFT-SAL-CHE-159	M	52	FTD	NA	Sporadic	France	31
DFT-SAL-PON-041	M	53	FTD	NA	Sporadic	France	31
DFT-DIJ-GAU-602	M	56	FTD	NA	Sporadic	France	31
DFT-NAN-CHE-135	M	57	FTD	NA	Familial	France	31

DFT-NAN-VIN-197	M	58	FTD	NA	Familial	France	31
DFT-REN-LAI-083	M	58	FTD	NA	Familial	France	31
A-ALZ-LIG-091	M	59	FTD	Cognitive	Familial	France	31
DFT-REN-MEG-193	M	59	FTD	Cognitive	Familial	France	31
DFT-SAL-GIL-045	M	59	FTD	Cognitive	Sporadic	France	31
DFT-SAL-PEY-622	M	65	FTD	Cognitive	Familial	France	31
DFT-SAL-COU-094	M	65	FTD	NA	Familial	France	31
FTD009	M	67	FTD	Cognitive	Sporadic	France	31
SAL-BOU-504	M	69	FTD	Cognitive	Familial	France	31
DFT-SAL-CAR-518	M	72	FTD	NA	Sporadic	France	31
DFT-STR-THE-119	M	72	FTD	NA	Sporadic	France	31
W513	F	43	ALS	Spinal	Sporadic	Germany	31
W316	F	52	ALS	Spinal	Sporadic	Germany	31
WUR006	F	54	ALS	NA	Familial	Germany	31
WUR378	F	54	ALS	NA	Familial	Germany	31
WUR820	F	54	ALS	NA	Familial	Germany	31
WUR237	F	55	ALS	Bulbar	Familial	Germany	31
W408	F	57	ALS	Bulbar	Sporadic	Germany	31
WUR496	F	58	ALS	NA	Familial	Germany	31
W201	F	58	ALS	Spinal	Sporadic	Germany	31
W424	F	58	ALS	Spinal	Sporadic	Germany	31
WUR048	F	58	ALS	Spinal	Familial	Germany	31
WUR107	F	59	ALS	Bulbar	Familial	Germany	31
W078	F	59	ALS	Spinal	Sporadic	Germany	31
WUR624	F	65	ALS	NA	Familial	Germany	31
W410	F	67	ALS	Bulbar	Sporadic	Germany	31
WUR636	F	67	ALS	NA	Familial	Germany	31
WUR409	F	68	ALS	Bulbar	Familial	Germany	31
WUR773	F	71	ALS	NA	Familial	Germany	31
W066	F	72	ALS	Spinal	Sporadic	Germany	31
W160	F	79	ALS	Bulbar	Sporadic	Germany	31
WUR521	M	35	ALS	NA	Familial	Germany	31
WUR162	M	37	ALS	Bulbar	Familial	Germany	31
WUR621	M	44	ALS	NA	Familial	Germany	31
W301	M	44	ALS	Spinal	Sporadic	Germany	31
W127	M	45	ALS	Spinal	Sporadic	Germany	31
WUR452	M	45	ALS	Spinal	Familial	Germany	31
W285	M	50	ALS	Spinal	Sporadic	Germany	31
W223	M	54	ALS	Spinal	Sporadic	Germany	31
W071	M	55	ALS	Bulbar	Sporadic	Germany	31
W460	M	55	ALS	Bulbar	Sporadic	Germany	31
W165	M	56	ALS	Spinal	Sporadic	Germany	31
W042	M	59	ALS	Spinal	Sporadic	Germany	31
W267	M	59	ALS	Spinal	Sporadic	Germany	31
W553	M	59	ALS	Spinal	Sporadic	Germany	31
W177	M	60	ALS	Spinal	Sporadic	Germany	31
W222	M	66	ALS	Bulbar	Sporadic	Germany	31

W180	M	70	ALS	Bulbar	Sporadic	Germany	31
KT348	F	26	Control	NA	NA	Germany	31
KT041	F	35	Control	NA	NA	Germany	31
PS25	F	61	FTD	Cognitive	Familial	Germany	31
PS2026	F	64	FTD	Cognitive	Familial	Germany	31
PS2009	M	55	FTD	Cognitive	Familial	Germany	31
PS31	M	61	FTD	Cognitive	Familial	Germany	31
NC1	M	45	ALS	Spinal	Familial	Ireland	31
ISR4021	F	64	ALS	Spinal	Familial	Israel	31
ISR0026	M	56	ALS	Spinal	Familial	Israel	31
ISR2138	M	63	ALS	Spinal	Familial	Israel	31
SLA2011-247	F	36	ALS	Spinal	Familial	Italy	31
SLA2008-0394	F	44	ALS	Spinal	Sporadic	Italy	31
SLA2010-312	F	44	ALS	Spinal	Familial	Italy	31
SLA2010-468	F	45	ALS	Bulbar	Familial	Italy	31
IT_00523	F	46	ALS	Spinal	Sporadic	Italy	31
IT_15344	F	46	ALS	Spinal	Sporadic	Italy	31
SLA2009-224	F	50	ALS	Spinal	Familial	Italy	31
M151	F	51	ALS	Bulbar	Familial	Italy	31
M10743-FG	F	51	ALS	Spinal	Familial	Italy	31
IT_02229	F	52	ALS	Spinal	Sporadic	Italy	31
IT_09798	F	52	ALS	Spinal	Sporadic	Italy	31
SLA2010-527	F	55	ALS	Spinal	Familial	Italy	31
SLA2011-82	F	57	ALS	Spinal	Familial	Italy	31
SLA2010-043	F	59	ALS	Bulbar	Familial	Italy	31
SLA2010-135	F	59	ALS	Bulbar	Familial	Italy	31
SLA2010-320	F	59	ALS	Spinal	Familial	Italy	31
M153	F	61	ALS	Bulbar	Familial	Italy	31
IT_01267	F	63	ALS	Spinal	Sporadic	Italy	31
SLA2008-266	F	63	ALS	Bulbar	Familial	Italy	31
SLA2011-78	F	63	ALS	Bulbar	Familial	Italy	31
FALSRO3	F	64	ALS	Spinal	Familial	Italy	31
SLA2008-0106	F	65	ALS	Spinal	Familial	Italy	31
PO2007-1876	F	67	ALS	Bulbar	Sporadic	Italy	31
PO2007-2029	F	67	ALS	Bulbar	Sporadic	Italy	31
IT_20115	F	68	ALS	Bulbar	Sporadic	Italy	31
SLA2011-297	F	72	ALS	Spinal	Familial	Italy	31
PO2007-1352	M	35	ALS	Spinal	Familial	Italy	31
IT_10736	M	40	ALS	Spinal	Sporadic	Italy	31
M1594	M	44	ALS	Spinal	Familial	Italy	31
SLA2008-0386	M	45	ALS	Spinal	Familial	Italy	31
SLA2011-89	M	48	ALS	Spinal	Familial	Italy	31
SLA2009-0092	M	48	ALS	Spinal	Sporadic	Italy	31
SLA2009-45	M	50	ALS	Bulbar	Familial	Italy	31
SLA2011-55	M	50	ALS	Bulbar	Familial	Italy	31
IT_15358	M	52	ALS	Bulbar	Sporadic	Italy	31
PO2007-1872	M	52	ALS	Spinal	Sporadic	Italy	31

PO2007-0976	M	53	ALS	Bulbar	Sporadic	Italy	31
SLA2009-0307	M	54	ALS	Spinal	Familial	Italy	31
SLA2011-294	M	54	ALS	Spinal	Familial	Italy	31
SLA2011-292	M	57	ALS	Spinal	Familial	Italy	31
SLA2011-273	M	59	ALS	Bulbar	Familial	Italy	31
SLA2010-0200	M	60	ALS	Spinal	Familial	Italy	31
SLA2009-300	M	62	ALS	Bulbar	Familial	Italy	31
PO2007-291	M	62	ALS	Spinal	Familial	Italy	31
IT_20118	M	63	ALS	Spinal	Sporadic	Italy	31
13807-BT	M	63	ALS	Spinal	Familial	Italy	31
SLA2009-0301	M	65	ALS	Spinal	Familial	Italy	31
SLA2009-0086	M	66	ALS	Spinal	Sporadic	Italy	31
IT_02612	M	66	ALS	Spinal	Sporadic	Italy	31
SLA2009-106	M	66	ALS	Bulbar	Familial	Italy	31
IT_02404	M	68	ALS	Bulbar	Sporadic	Italy	31
SLA2011-289	M	69	ALS	Bulbar	Familial	Italy	31
IT_10744	M	70	ALS	Spinal	Sporadic	Italy	31
M210	F	49	ALS	Spinal	Familial	Japan	31
dutch2	F	40	FTD	NA	Familial	Netherlands	31
dutch3	F	40	FTD	NA	Familial	Netherlands	31
dutch6	F	42	FTD	NA	Familial	Netherlands	31
dutch9	F	45	FTD	NA	Familial	Netherlands	31
dutch17	F	51	FTD	NA	Familial	Netherlands	31
dutch20	F	51	FTD	NA	Familial	Netherlands	31
dutch30	F	54	FTD	NA	Familial	Netherlands	31
dutch215	F	58	FTD	NA	Sporadic	Netherlands	31
dutch248	F	60	FTD	NA	Sporadic	Netherlands	31
dutch59	F	60	FTD	NA	Familial	Netherlands	31
dutch67	F	61	FTD	NA	Familial	Netherlands	31
dutch77	F	63	FTD	NA	Familial	Netherlands	31
dutch279	F	63	FTD	NA	Sporadic	Netherlands	31
dutch84	F	63	FTD	NA	Familial	Netherlands	31
dutch86	F	64	FTD	NA	Familial	Netherlands	31
dutch93	F	65	FTD	NA	Familial	Netherlands	31
dutch100	F	66	FTD	NA	Familial	Netherlands	31
dutch329	F	70	FTD	NA	Sporadic	Netherlands	31
dutch4	M	40	FTD	NA	Familial	Netherlands	31
dutch13	M	49	FTD	NA	Familial	Netherlands	31
dutch24	M	52	FTD	NA	Familial	Netherlands	31
dutch35	M	55	FTD	NA	Familial	Netherlands	31
dutch36	M	55	FTD	NA	Familial	Netherlands	31
dutch193	M	56	FTD	NA	Sporadic	Netherlands	31
dutch38	M	56	FTD	NA	Familial	Netherlands	31
dutch44	M	57	FTD	NA	Familial	Netherlands	31
dutch49	M	58	FTD	NA	Familial	Netherlands	31
dutch57	M	60	FTD	NA	Familial	Netherlands	31
dutch61	M	60	FTD	NA	Familial	Netherlands	31

dutch62	M	60	FTD	NA	Familial	Netherlands	31
dutch66	M	61	FTD	NA	Familial	Netherlands	31
dutch68	M	61	FTD	NA	Familial	Netherlands	31
dutch76	M	63	FTD	NA	Familial	Netherlands	31
dutch102	M	67	FTD	NA	Familial	Netherlands	31
dutch116	M	76	FTD	NA	Familial	Netherlands	31
SLA2011-207	F	34	ALS	Spinal	Sporadic	Sardinia	31
15344-FV	F	46	ALS	Spinal	Familial	Sardinia	31
SLA2010-066	F	55	ALS	Spinal	Familial	Sardinia	31
SLA2011-228	F	57	ALS	Bulbar	Familial	Sardinia	31
SLA2010-506	F	57	ALS	Spinal	Sporadic	Sardinia	31
SLA2011-165	F	60	ALS	Spinal	Familial	Sardinia	31
SR1	F	61	ALS	Bulbar	Sporadic	Sardinia	31
SA1	F	67	ALS	Bulbar	Sporadic	Sardinia	31
SLA2009-231	M	35	ALS	Bulbar	Sporadic	Sardinia	31
SLA2011-240	M	50	ALS	Spinal	Familial	Sardinia	31
SLA2010-517	M	54	ALS	Spinal	Sporadic	Sardinia	31
SLA2009-211	M	56	ALS	Spinal	Familial	Sardinia	31
SLA2011-226	M	58	ALS	Spinal	Sporadic	Sardinia	31
SLA2009-205	M	60	ALS	Spinal	Sporadic	Sardinia	31
SLA2009-204	M	62	ALS	Bulbar	Familial	Sardinia	31
SLA2010-524	M	62	ALS	Spinal	Sporadic	Sardinia	31
SLA2009-191	M	63	ALS	Spinal	Sporadic	Sardinia	31
SLA2009-195	M	66	ALS	Bulbar	Familial	Sardinia	31
SLA2009-125	M	68	ALS	Spinal	Familial	Sardinia	31
SLA2009-190	M	69	ALS	Spinal	Familial	Sardinia	31
SLA2009-162	M	78	ALS	Bulbar	Familial	Sardinia	31
FTD2011-2	M	60	FTD	Cognitive	Familial	Sardinia	31
M3962-3963	F	68	FTD	Cognitive	Familial	Sweden	31
M9518	F	45	FTD	Cognitive	Familial	Taiwan	31
M9428	F	54	FTD	Cognitive	Familial	Taiwan	31
BM018	F	50	ALS	Bulbar	Familial	UK	31
BM009	F	52	ALS	Spinal	Sporadic	UK	31
BM032	F	69	ALS	Spinal	Sporadic	UK	31
BM043	F	71	ALS	Bulbar	Sporadic	UK	31
BM156	M	40	ALS	Spinal	Sporadic	UK	31
BM065	M	51	ALS	Bulbar	Sporadic	UK	31
BM014	M	51	ALS	Bulbar	Familial	UK	31
BM139	M	58	ALS	Spinal	Sporadic	UK	31
BM074	M	58	ALS	Spinal	Familial	UK	31
BM110	M	62	ALS	Spinal	Familial	UK	31
BM151	M	63	ALS	Spinal	Familial	UK	31
M4017	F	45	ALS	Spinal	Familial	UK	31
M10456	F	53	ALS	Spinal	Familial	UK	31
M11159	F	53	ALS	Spinal	Sporadic	UK	31
M9001	F	60	ALS	Spinal	Familial	UK	31
M11135	F	64	ALS	Spinal	Familial	UK	31

M11171	F	68	ALS	Bulbar	Sporadic	UK	31
M11125	M	37	ALS	Spinal	Sporadic	UK	31
M11184	M	46	ALS	Spinal	Sporadic	UK	31
M11083	M	59	ALS	Spinal	Familial	UK	31
M10325	F	44	FTD	Cognitive	Familial	UK	31
M4024	F	51	FTD	Cognitive	Familial	UK	31
M8326	M	51	FTD	Cognitive	Familial	UK	31
M10322	M	73	FTD	Cognitive	Sporadic	UK	31
M423	F	47	FTD	NA	Familial	UK	31
M290	F	52	FTD	NA	Familial	UK	31
M441	F	53	FTD	Cognitive	Sporadic	UK	31
M31198996	F	54	FTD	NA	Familial	UK	31
M416	F	55	FTD	NA	Familial	UK	31
M343	F	60	FTD	NA	Familial	UK	31
M344	F	60	FTD	NA	Familial	UK	31
M553	F	61	FTD	Cognitive	Sporadic	UK	31
M31200871	F	62	FTD	Cognitive	Sporadic	UK	31
M231	F	67	FTD	Cognitive	Sporadic	UK	31
M31200941	F	68	FTD	Cognitive	Sporadic	UK	31
M31200082	M	39	FTD	NA	Sporadic	UK	31
M31200979	M	45	FTD	Cognitive	Sporadic	UK	31
M539	M	49	FTD	NA	Familial	UK	31
M336	M	56	FTD	Cognitive	Sporadic	UK	31
M31199512	M	56	FTD	Cognitive	Sporadic	UK	31
M397	M	57	FTD	NA	Familial	UK	31
M375	M	58	FTD	Cognitive	Sporadic	UK	31
M546	M	58	FTD	Cognitive	Sporadic	UK	31
M31200646	M	58	FTD	Cognitive	Sporadic	UK	31
M251	M	60	FTD	Cognitive	Sporadic	UK	31
M31200287	M	62	FTD	Cognitive	Sporadic	UK	31
M31200050	M	63	FTD	Cognitive	Sporadic	UK	31
M119	M	63	FTD	NA	Familial	UK	31
M31200523	M	65	FTD	Cognitive	Sporadic	UK	31
M155	M	68	FTD	Cognitive	Sporadic	UK	31
M74	M	70	FTD	Cognitive	Sporadic	UK	31
M4726	F	46	FTD	Cognitive	Familial	UK	31
M12440	F	50	FTD	Cognitive	Familial	UK	31
M12533	F	53	FTD	Cognitive	Familial	UK	31
M12417	F	54	FTD	Cognitive	Sporadic	UK	31
M20784	F	56	FTD	Cognitive	Familial	UK	31
M25551	F	56	FTD	Cognitive	Familial	UK	31
M6569	F	63	FTD	Cognitive	Familial	UK	31
M19240	F	68	FTD	Cognitive	Familial	UK	31
M22415	M	39	FTD	Cognitive	Familial	UK	31
M16036	M	48	FTD	Cognitive	Sporadic	UK	31
M12368	M	51	FTD	Cognitive	Familial	UK	31
M12458	M	54	FTD	Cognitive	Familial	UK	31

M1831-A138	M	55	FTD	Cognitive	Familial	UK	31
M12475	M	55	FTD	Cognitive	Sporadic	UK	31
M25478	M	58	FTD	Cognitive	Familial	UK	31
M17323	M	59	FTD	Cognitive	Familial	UK	31
M20737	M	59	FTD	Cognitive	Sporadic	UK	31
M19245	M	62	FTD	Cognitive	Sporadic	UK	31
M5765	F	50	ALS	Spinal	Sporadic	UK	31
M6282	F	56	ALS	Bulbar	Familial	UK	31
M5795	F	57	ALS	Bulbar	Familial	UK	31
M5664	F	58	ALS	Spinal	Familial	UK	31
M5803	F	58	ALS	Spinal	Sporadic	UK	31
M5656	F	59	ALS	Spinal	Sporadic	UK	31
M5777	F	77	ALS	Spinal	Familial	UK	31
M5769	M	34	ALS	Spinal	Sporadic	UK	31
M6258	M	55	ALS	Bulbar	Familial	UK	31
NP208-08_C	M	55	ALS	NA	Familial	UK	31
M6219	M	57	ALS	Spinal	Sporadic	UK	31
M6222	M	58	ALS	Cognitive	Sporadic	UK	31
M399	F	69	FTD	Cognitive	Sporadic	UK	31
M319	M	48	FTD	Cognitive	Familial	UK	31
LDP0474	F	27	ALS	Bulbar	Familial	UK	31
LDP0257	F	36	ALS	Bulbar	Familial	UK	31
LDP0456	F	45	ALS	Bulbar	Familial	UK	31
LDP0035	F	48	ALS	Spinal	Familial	UK	31
LDP0092	F	48	ALS	Spinal	Familial	UK	31
M133	F	49	ALS	Spinal	Sporadic	UK	31
LDP0442	F	50	ALS	Bulbar	Familial	UK	31
M2	F	52	ALS	Spinal	Sporadic	UK	31
M143	F	56	ALS	Spinal	Familial	UK	31
LDP0793	F	57	ALS	Bulbar	Sporadic	UK	31
M115	F	57	ALS	Spinal	Sporadic	UK	31
M14	F	58	ALS	Spinal	Sporadic	UK	31
M51	F	59	ALS	Spinal	Familial	UK	31
M37	F	59	ALS	Spinal	Sporadic	UK	31
LDP0340	F	61	ALS	Bulbar	Familial	UK	31
M70	F	61	ALS	Bulbar	Sporadic	UK	31
M171	F	61	ALS	Spinal	Familial	UK	31
LDP0163	F	61	ALS	Spinal	Familial	UK	31
M104	F	61	ALS	Spinal	Sporadic	UK	31
M136	F	62	ALS	Bulbar	Familial	UK	31
LDP0216	F	62	ALS	Spinal	Sporadic	UK	31
LDP0632	F	63	ALS	Bulbar	Sporadic	UK	31
M164	F	63	ALS	Cognitive	Familial	UK	31
LDP0909	F	63	ALS	Spinal	Familial	UK	31
LDP0039	F	65	ALS	Bulbar	Sporadic	UK	31
M25	F	66	ALS	Bulbar	Sporadic	UK	31
M13	F	66	ALS	Spinal	Sporadic	UK	31

LDP0284	F	66	ALS	Spinal	Sporadic	UK	31
LDP0221	F	67	ALS	Spinal	Sporadic	UK	31
M168	F	71	ALS	Spinal	Sporadic	UK	31
LDP0208	F	71	ALS	Spinal	Sporadic	UK	31
LDP0183	M	30	ALS	Bulbar	Sporadic	UK	31
M92	M	42	ALS	Spinal	Familial	UK	31
M12	M	43	ALS	Spinal	Sporadic	UK	31
M128	M	45	ALS	Spinal	Sporadic	UK	31
M155	M	47	ALS	Spinal	Familial	UK	31
LDP1156	M	48	ALS	Bulbar	Familial	UK	31
LDP0312	M	50	ALS	Spinal	Familial	UK	31
LDP0285	M	51	ALS	Bulbar	Sporadic	UK	31
LDP0870	M	55	ALS	Spinal	Sporadic	UK	31
LDP0639	M	56	ALS	Bulbar	Sporadic	UK	31
M158	M	56	ALS	Spinal	Sporadic	UK	31
LDP0317	M	59	ALS	Spinal	Sporadic	UK	31
LDP0399	M	60	ALS	Bulbar	Sporadic	UK	31
LDP0955	M	60	ALS	Bulbar	Sporadic	UK	31
M114	M	60	ALS	Spinal	Sporadic	UK	31
M118	M	60	ALS	Spinal	Sporadic	UK	31
M110	M	62	ALS	Bulbar	Sporadic	UK	31
M17	M	62	ALS	Spinal	Familial	UK	31
M48	M	63	ALS	Spinal	Sporadic	UK	31
M65	M	63	ALS	Spinal	Sporadic	UK	31
LDP0837	M	64	ALS	Bulbar	Sporadic	UK	31
M101	M	64	ALS	Spinal	Familial	UK	31
M23	M	64	ALS	Spinal	Sporadic	UK	31
M120	M	64	ALS	Spinal	Sporadic	UK	31
M167	M	67	ALS	Spinal	Familial	UK	31
M170	M	69	ALS	Spinal	Familial	UK	31
M165	M	76	ALS	Spinal	Sporadic	UK	31
AR240	F	55	ALS	Bulbar	Familial	UK	31
Richard_ALS_152	F	56	ALS	Spinal	Sporadic	UK	31
Richard_ALS_100	F	68	ALS	Spinal	Sporadic	UK	31
AR252	M	48	ALS	Spinal	Sporadic	UK	31
AR149	M	58	ALS	Spinal	Familial	UK	31
Richard_ALS_106	M	66	ALS	Spinal	Sporadic	UK	31
M1042	F	68	ALS	Bulbar	Sporadic	US	31
M1614	F	68	ALS	Bulbar	Sporadic	US	31
M1271	F	58	ALS	Bulbar	Sporadic	US	31
M1500	F	69	ALS	Bulbar	Sporadic	US	31
M1667	F	71	ALS	Spinal	Sporadic	US	31
M1461	M	49	ALS	Spinal	Sporadic	US	31
M1641	M	58	ALS	Bulbar	Sporadic	US	31
M2106	M	61	ALS	Bulbar	Sporadic	US	31
ND09438	F	43	ALS	Spinal	Sporadic	US	31
ND11933	F	44	ALS	Bulbar	Familial	US	31

ND12526	F	45	ALS	Spinal	Sporadic	US	31
ERG7311	F	46	ALS	Bulbar	Familial	US	31
ND12277	F	46	ALS	Bulbar	Familial	US	31
ND12909	F	49	ALS	Bulbar	Familial	US	31
ND10689	F	49	ALS	Spinal	Familial	US	31
ND12105	F	49	ALS	Spinal	Sporadic	US	31
B004	F	50	ALS	Bulbar	Sporadic	US	31
ND10000	F	50	ALS	Spinal	Sporadic	US	31
ND10828	F	51	ALS	Spinal	Sporadic	US	31
ND10579	F	52	ALS	Bulbar	Sporadic	US	31
2209-0009	F	54	ALS	Spinal	Familial	US	31
ND09362	F	54	ALS	Spinal	Sporadic	US	31
B035	F	55	ALS	Bulbar	Sporadic	US	31
2013-0100	F	55	ALS	Spinal	Familial	US	31
ND11374	F	55	ALS	Spinal	Familial	US	31
ND09576	F	55	ALS	Spinal	Sporadic	US	31
ND12421	F	55	ALS	Spinal	Sporadic	US	31
ERG9106	F	56	ALS	Bulbar	Familial	US	31
ND13803	F	57	ALS	Bulbar	Familial	US	31
2209-0023	F	57	ALS	Spinal	Sporadic	US	31
B079	F	58	ALS	NA	Sporadic	US	31
ND10905	F	58	ALS	Spinal	Familial	US	31
ND12199	F	59	ALS	Spinal	Sporadic	US	31
ND12089	F	60	ALS	Bulbar	Familial	US	31
ND13615	F	60	ALS	Bulbar	Sporadic	US	31
ND09204	F	60	ALS	Spinal	Familial	US	31
ND08544	F	61	ALS	Bulbar	Familial	US	31
ND11081	F	61	ALS	Bulbar	Familial	US	31
ND15686	F	62	ALS	Spinal	Familial	US	31
B063	F	63	ALS	NA	Sporadic	US	31
ND12334	F	63	ALS	Spinal	Sporadic	US	31
ND14911	F	64	ALS	Spinal	Familial	US	31
ND20142	F	65	ALS	Spinal	Familial	US	31
ND09996	F	66	ALS	Spinal	Sporadic	US	31
ND11917	F	67	ALS	Bulbar	Familial	US	31
ND11680	F	68	ALS	Bulbar	Familial	US	31
ND12754	F	68	ALS	Bulbar	Familial	US	31
ND08980	F	70	ALS	Spinal	Sporadic	US	31
ND14339	F	71	ALS	Bulbar	Familial	US	31
ND14596	F	72	ALS	Spinal	Familial	US	31
ERG7045	M	37	ALS	Spinal	Familial	US	31
ND15362	M	37	ALS	Spinal	Familial	US	31
2013-0089	M	44	ALS	Bulbar	Familial	US	31
B029	M	44	ALS	Spinal	Familial	US	31
ND11343	M	44	ALS	Spinal	Sporadic	US	31
ND12904	M	45	ALS	Spinal	Familial	US	31
ND08957	M	46	ALS	Spinal	Familial	US	31

ND11252	M	47	ALS	Spinal	Familial	US	31
ND12099	M	48	ALS	Spinal	Familial	US	31
B055	M	48	ALS	Spinal	Sporadic	US	31
ND10284	M	49	ALS	Spinal	Familial	US	31
ND06751	M	50	ALS	Spinal	Familial	US	31
ND09492	M	51	ALS	Spinal	Familial	US	31
ND12402	M	52	ALS	Spinal	Familial	US	31
ND12161	M	52	ALS	Spinal	Sporadic	US	31
ND11411	M	53	ALS	Bulbar	Familial	US	31
ND12100	M	55	ALS	Bulbar	Familial	US	31
ERG8863	M	55	ALS	Spinal	Familial	US	31
ND10123	M	55	ALS	Spinal	Sporadic	US	31
ND09373	M	56	ALS	NA	Familial	US	31
ND11494	M	56	ALS	Spinal	Familial	US	31
ND14442	M	56	ALS	Spinal	Familial	US	31
ND11583	M	57	ALS	Bulbar	Familial	US	31
ND09508	M	57	ALS	Spinal	Familial	US	31
2122-0004	M	57	ALS	Spinal	Sporadic	US	31
ERG9090	M	58	ALS	Spinal	Familial	US	31
ND13682	M	58	ALS	Spinal	Familial	US	31
ND13828	M	58	ALS	Spinal	Familial	US	31
ND11869	M	58	ALS	Spinal	Sporadic	US	31
ND14954	M	59	ALS	Bulbar	Familial	US	31
ND10101	M	60	ALS	Spinal	Familial	US	31
ND10023	M	60	ALS	Spinal	Sporadic	US	31
B048	M	61	ALS	NA	Sporadic	US	31
2209-0007	M	61	ALS	Spinal	Familial	US	31
B062	M	62	ALS	NA	Sporadic	US	31
ND10973	M	63	ALS	Spinal	Sporadic	US	31
B042	M	64	ALS	Spinal	Familial	US	31
ND10966	M	64	ALS	Spinal	Familial	US	31
ND14186	M	64	ALS	Spinal	Familial	US	31
2013-0011	M	65	ALS	Spinal	Familial	US	31
ND11749	M	65	ALS	Spinal	Familial	US	31
B016	M	66	ALS	Bulbar	Sporadic	US	31
ND07489	M	66	ALS	Spinal	Familial	US	31
ND11548	M	67	ALS	Spinal	Familial	US	31
ND15814	M	68	ALS	Spinal	Familial	US	31
2013-0094	M	71	ALS	Spinal	Familial	US	31
B075	M	73	ALS	NA	Sporadic	US	31
B057	M	74	ALS	NA	Sporadic	US	31
ND09377	M	77	ALS	Spinal	Sporadic	US	31
B061	M	80	ALS	NA	Sporadic	US	31
ND12455	M	80	ALS	Spinal	Familial	US	31
ND15567	F	36	Control	NA	NA	US	31
M105957	F	41	ALS	Spinal	Sporadic	US	31
M110368	F	41	ALS	Spinal	Sporadic	US	31

M101448	F	46	ALS	Bulbar	Sporadic	US	31
M114747	F	52	ALS	Spinal	Sporadic	US	31
M110243	F	65	ALS	Spinal	Sporadic	US	31
M107297	F	67	ALS	Spinal	Sporadic	US	31
M105812	M	40	ALS	NA	Sporadic	US	31
M106599	M	43	ALS	Spinal	Sporadic	US	31
M113381	M	49	ALS	Spinal	Sporadic	US	31
M114327	M	51	ALS	Bulbar	Sporadic	US	31
M105547	M	55	ALS	Bulbar	Sporadic	US	31
M100089	M	60	ALS	Spinal	Sporadic	US	31
M100737	M	66	ALS	Bulbar	Sporadic	US	31
M104236	M	71	ALS	Spinal	Sporadic	US	31
Mandic1	F	64	FTD	Cognitive	NA	Serbia	32
Mandic2	F	50	FTD	Cognitive	NA	Serbia	32
Mandic3	F	53	FTD	Cognitive	NA	Serbia	32
Mandic4	F	41	ALS-FTD	Bulbar	NA	Serbia	32
Millecamps1	M	68	ALS	Spinal	Familial	France	33
Millecamps2	M	68	ALS	Bulbar	Familial	France	33
Millecamps3	M	49	ALS	Spinal	Familial	France	33
Millecamps4	M	45	ALS	Spinal	Familial	France	33
Millecamps5	M	59	ALS	Bulbar	Familial	France	33
Millecamps6	M	46	ALS-FTD	Spinal	Familial	France	33
Millecamps7	M	62	ALS	Bulbar	Familial	France	33
Millecamps8	M	70	ALS-FTD	Bulbar	Familial	France	33
Millecamps9	F	58	ALS	Bulbar	Familial	France	33
Millecamps10	M	49	ALS-FTD	Spinal	Familial	France	33
Millecamps11	M	47	ALS-FTD	Bulbar	Familial	France	33
Millecamps12	M	61	ALS	Bulbar	Familial	France	33
Millecamps13	M	52	ALS	Spinal	Familial	France	33
Millecamps14	M	71	ALS	Spinal	Familial	France	33
Millecamps15	F	44	ALS	Spinal	Familial	France	33
Millecamps16	F	64	ALS	Spinal	Familial	France	33
Millecamps17	M	53	ALS	Bulbar	Familial	France	33
Millecamps18	F	71	ALS-FTD	Bulbar	Familial	France	33
Millecamps19	F	70	ALS-FTD	Spinal	Familial	France	33
Millecamps20	F	63	ALS	Spinal	Familial	France	33
Millecamps21	M	62	ALS	Spinal	Familial	France	33
Millecamps22	M	50	ALS	Spinal	Familial	France	33
Millecamps23	M	44	ALS	Spinal	Familial	France	33
Millecamps24	M	75	ALS-FTD	Bulbar	Familial	France	33
Millecamps25	M	48	ALS	Bulbar	Familial	France	33
Millecamps26	M	67	ALS-FTD	NA	Familial	France	33
Millecamps27	F	60	ALS	Spinal	Familial	France	33
Millecamps28	F	70	ALS	Spinal	Familial	France	33
Millecamps29	F	52	ALS	Bulbar	Familial	France	33
Millecamps30	F	58	ALS-FTD	Spinal	Familial	France	33
Millecamps31	M	69	ALS	Spinal	Familial	France	33

Millecamps32	M	63	ALS	Bulbar	Familial	France	33
Millecamps33	M	61	ALS	Spinal	Familial	France	33
Millecamps34	M	56	ALS	Bulbar	Familial	France	33
Millecamps35	M	64	ALS	Bulbar	Familial	France	33
Millecamps36	F	52	ALS	Bulbar	Familial	France	33
Millecamps37	F	88	Control	NA	NA	France	33
Mok1	M	65	ALS	NA	Sporadic	Greece	34
Mok2	M	57	ALS	NA	Sporadic	Greece	34
Mok3	M	58	ALS	NA	Sporadic	Greece	34
Mok4	F	73	ALS	NA	Familial	Greece	34
Mok5	M	56	ALS	NA	Sporadic	Greece	34
Mok6	F	61	ALS	NA	Familial	Greece	34
Mok7	M	25	ALS	NA	Sporadic	Greece	34
Mok8	F	70	ALS	NA	Sporadic	Greece	34
Mok9	M	45	ALS	NA	Sporadic	Greece	34
Mok10	M	48	ALS	NA	Sporadic	Greece	34
Mok11	M	55	ALS	NA	Sporadic	Greece	34
Mok12	M	54	ALS	NA	Sporadic	Greece	34
Mok13	M	70	ALS	NA	Sporadic	Greece	34
Mok14	F	46	ALS	NA	Familial	Greece	34
Mok15	M	55	ALS	NA	Familial	Greece	34
Mok16	M	43	ALS	NA	Familial	Greece	34
Murray1	F	48	ALS	Spinal	NA	US	35
Murray2	F	47	ALS	Bulbar	NA	US	35
Murray3	M	52	ALS	Spinal	NA	US	35
Murray4	F	61	ALS	Bulbar	NA	US	35
Murray5	F	76	ALS	NA	NA	US	35
Murray6	M	56	ALS	General	NA	US	35
Murray7	M	56	ALS-FTD	General	NA	US	35
Murray8	M	52	ALS	General	NA	US	35
Murray9	F	50	FTD	NA	NA	US	35
Murray10	F	57	FTD	NA	NA	US	35
Murray11	M	57	FTD	NA	NA	US	35
Murray12	F	54	FTD	NA	NA	US	35
Murray13	F	70	FTD	NA	NA	US	35
Murray14	M	66	FTD	NA	NA	US	35
Murray15	M	70	FTD	NA	NA	US	35
Murray16	M	69	FTD	NA	NA	US	35
Murray17	M	63	FTD	NA	NA	US	35
Murray18	M	74	FTD	NA	NA	US	35
Murray19	F	79	FTD	NA	NA	US	35
Murray20	M	78	FTD	NA	NA	US	35
Ogaki1	M	65	ALS-FTD	Spinal	Familial	Japan	36
Ogaki2	M	57	ALS	Bulbar	Familial	Japan	36
Ogaki3	M	72	ALS	Spinal	Sporadic	Japan	36
Ozoguz1	M	32	ALS	Spinal	Familial	Turkey	37
Ozoguz2	F	46	ALS	NA	Familial	Turkey	37

Ozoguz3	M	46	ALS	Bulbar	Familial	Turkey	37
Ozoguz4	M	56	ALS	Spinal	Familial	Turkey	37
Ozoguz5	M	80	ALS	Spinal	Familial	Turkey	37
Ozoguz6	M	63	ALS	Spinal	Familial	Turkey	37
Ozoguz7	F	37	ALS	Spinal	Familial	Turkey	37
Ozoguz8	F	62	ALS	Spinal	Familial	Turkey	37
Ozoguz9	F	58	ALS	Spinal	Familial	Turkey	37
Ozoguz10	F	57	ALS	NA	Familial	Turkey	37
Ozoguz11	M	58	ALS	Spinal	Familial	Turkey	37
Ozoguz12	M	46	ALS	Bulbar	Familial	Turkey	37
Ozoguz13	F	53	ALS	Bulbar	Familial	Turkey	37
Ozoguz14	F	63	ALS	Bulbar	Familial	Turkey	37
Ozoguz15	M	71	ALS	Spinal	Familial	Turkey	37
Ozoguz16	M	54	ALS	Spinal	Familial	Turkey	37
Ozoguz17	F	63	ALS	Spinal	Familial	Turkey	37
Ozoguz18	M	62	ALS	Spinal	Familial	Turkey	37
Ozoguz19	F	69	ALS	Spinal	Sporadic	Turkey	37
Ozoguz20	M	48	ALS	NA	Sporadic	Turkey	37
Ozoguz21	M	49	ALS	NA	Sporadic	Turkey	37
Ozoguz22	F	48	ALS	General	Sporadic	Turkey	37
Ozoguz23	F	55	ALS	Spinal	Sporadic	Turkey	37
Ozoguz24	M	42	ALS	Spinal	Sporadic	Turkey	37
Ozoguz25	F	52	ALS	Spinal	Sporadic	Turkey	37
Ozoguz26	M	53	ALS	Spinal	Sporadic	Turkey	37
Ozoguz27	M	56	ALS	General	Sporadic	Turkey	37
Ozoguz28	M	69	ALS	Bulbar	Sporadic	Turkey	37
Ozoguz29	M	60	ALS	Spinal	Sporadic	Turkey	37
Pamphlett1	M	36	ALS	NA	Sporadic	Australia	38
Pamphlett2	M	46	ALS	NA	Sporadic	Australia	38
Sabatelli1	M	52	ALS-FTD	Bulbar	Sporadic	Italy	39
Sabatelli2	M	65	ALS	Spinal	Sporadic	Italy	39
Sabatelli3	M	68	ALS	Bulbar	Sporadic	Italy	39
Sabatelli4	M	62	ALS-FTD	Spinal	Sporadic	Italy	39
Sabatelli5	M	53	ALS-FTD	Bulbar	Sporadic	Italy	39
Sabatelli6	M	70	ALS	Spinal	Sporadic	Italy	39
Sabatelli7	F	51	ALS	Spinal	Sporadic	Italy	39
Sabatelli8	M	65	ALS	Spinal	Sporadic	Italy	39
Sabatelli9	M	48	ALS	Spinal	Sporadic	Italy	39
Sabatelli10	F	52	ALS-FTD	Spinal	Sporadic	Italy	39
Sabatelli11	F	66	ALS	Bulbar	Sporadic	Italy	39
Sabatelli12	M	52	ALS-FTD	Spinal	Sporadic	Italy	39
Sabatelli13	F	43	ALS-FTD	Spinal	Sporadic	Italy	39
Sabatelli14	M	40	ALS	Spinal	Sporadic	Italy	39
Sabatelli15	F	41	ALS	Bulbar	Sporadic	Italy	39
Sabatelli16	F	72	ALS-FTD	Spinal	Sporadic	Italy	39
Sabatelli17	F	60	ALS-FTD	Bulbar	Sporadic	Italy	39
Sabatelli18	F	62	ALS	Spinal	Sporadic	Italy	39

Sabatelli19	M	69	ALS-FTD	Bulbar	Sporadic	Italy	39
Sabatelli20	F	78	ALS-FTD	Bulbar	Sporadic	Italy	39
Sabatelli21	M	56	ALS	Spinal	Sporadic	Italy	39
Sabatelli22	M	83	ALS-FTD	Bulbar	Sporadic	Italy	39
Sabatelli23	F	32	ALS	Spinal	Sporadic	Italy	39
Sabatelli24	F	34	ALS-FTD	Spinal	Sporadic	Italy	39
Sabatelli25	M	58	ALS-FTD	Spinal	Sporadic	Italy	39
Sabatelli26	M	54	ALS	Spinal	Sporadic	Italy	39
Sabatelli27	F	63	ALS-FTD	Spinal	Sporadic	Italy	39
Sabatelli28	M	63	ALS-FTD	Spinal	Sporadic	Italy	39
Sabatelli29	M	60	ALS	Spinal	Sporadic	Italy	39
Sabatelli30	M	62	ALS-FTD	Spinal	Sporadic	Italy	39
Sabatelli31	F	67	ALS-FTD	Bulbar	Sporadic	Italy	39
Sabatelli32	F	67	ALS-FTD	Bulbar	Sporadic	Italy	39
Sabatelli33	F	68	ALS	Bulbar	Sporadic	Italy	39
Sabatelli34	M	75	ALS	Bulbar	Sporadic	Italy	39
Sabatelli35	F	59	ALS	Spinal	Sporadic	Italy	39
Sabatelli36	M	60	ALS	Spinal	Sporadic	Italy	39
Sabatelli37	F	65	ALS	Bulbar	Sporadic	Italy	39
Sabatelli38	M	61	ALS	Spinal	Sporadic	Italy	39
Sabatelli39	F	38	ALS	Spinal	Sporadic	Italy	39
Sabatelli40	F	42	ALS	Spinal	Sporadic	Italy	39
Sabatelli41	M	44	ALS	Spinal	Sporadic	Italy	39
Sabatelli42	M	68	ALS	Spinal	Sporadic	Italy	39
Sabatelli43	M	64	ALS-FTD	Spinal	Sporadic	Italy	39
Sabatelli44	F	43	ALS	Spinal	Sporadic	Italy	39
Sabatelli45	F	49	ALS	Bulbar	Sporadic	Italy	39
Sabatelli46	F	50	ALS	Spinal	Sporadic	Italy	39
Sabatelli47	F	58	ALS	Spinal	Sporadic	Italy	39
Sabatelli48	F	44	ALS	Spinal	Sporadic	Italy	39
Sabatelli49	M	59	ALS	Bulbar	Sporadic	Italy	39
Sabatelli50	M	48	ALS	Spinal	Sporadic	Italy	39
Sabatelli51	M	51	ALS	Spinal	Sporadic	Italy	39
Sabatelli52	M	62	ALS	Spinal	Sporadic	Italy	39
Sabatelli53	F	43	ALS	Bulbar	Sporadic	Italy	39
Sabatelli54	M	36	ALS	Spinal	Sporadic	Italy	39
Sabatelli55	F	66	ALS-FTD	Spinal	Sporadic	Italy	39
Sabatelli56	M	58	ALS-FTD	Spinal	Sporadic	Italy	39
Sabatelli57	F	59	ALS-FTD	Bulbar	Sporadic	Italy	39
Sabatelli58	F	57	ALS	Bulbar	Sporadic	Italy	39
Sabatelli59	F	67	ALS	Spinal	Sporadic	Italy	39
Sabatelli60	F	59	ALS	Spinal	Sporadic	Italy	39
Sabatelli61	M	50	ALS	Spinal	Sporadic	Italy	39
Sabatelli62	F	71	ALS-FTD	Bulbar	Sporadic	Italy	39
Sabatelli63	F	67	ALS	Bulbar	Sporadic	Italy	39
Sabatelli64	M	43	ALS	Spinal	Sporadic	Italy	39
Sabatelli65	M	56	ALS	Spinal	Sporadic	Italy	39

Sabatelli66	M	69	ALS	Bulbar	Sporadic	Italy	39
Sabatelli67	F	62	ALS	Spinal	Sporadic	Italy	39
Sabatelli68	F	56	ALS	Spinal	Sporadic	Italy	39
Sabatelli69	M	49	ALS-FTD	Bulbar	Sporadic	Italy	39
Snowden1	F	54	ALS-FTD	Bulbar	Sporadic	UK	40
Snowden2	F	54	ALS-FTD	Bulbar	Familial	UK	40
Snowden3	F	72	ALS-FTD	Bulbar	Sporadic	UK	40
Snowden4	M	58	ALS-FTD	Bulbar	NA	UK	40
Snowden5	M	57	ALS-FTD	Spinal	Sporadic	UK	40
Snowden6	F	68	ALS-FTD	Bulbar	Familial	UK	40
Snowden7	F	70	ALS-FTD	General	Familial	UK	40
Snowden8	M	58	ALS-FTD	General	Sporadic	UK	40
Snowden9	M	57	ALS-FTD	General	Familial	UK	40
Snowden10	M	49	FTD	NA	Familial	UK	40
Snowden11	M	55	FTD	NA	Familial	UK	40
Snowden12	M	64	FTD	NA	Familial	UK	40
Snowden13	M	65	FTD	NA	Familial	UK	40
Snowden14	F	53	FTD	NA	Sporadic	UK	40
Snowden15	M	58	FTD	NA	Sporadic	UK	40
Snowden16	M	62	FTD	NA	Familial	UK	40
Snowden17	M	68	FTD	NA	Sporadic	UK	40
Snowden18	M	39	FTD	NA	Familial	UK	40
Snowden19	M	70	FTD	NA	Sporadic	UK	40
Snowden20	F	52	FTD	NA	Familial	UK	40
Snowden21	M	55	FTD	NA	Familial	UK	40
Snowden22	F	62	FTD	NA	Familial	UK	40
Snowden23	M	46	FTD	NA	Familial	UK	40
Snowden24	M	59	FTD	NA	Sporadic	UK	40
Snowden25	M	55	FTD	NA	Sporadic	UK	40
Snowden26	F	72	FTD	NA	Sporadic	UK	40
Snowden27	M	56	FTD	NA	Familial	UK	40
Snowden28	F	60	FTD	NA	Familial	UK	40
Snowden29	F	47	FTD	NA	Familial	UK	40
Snowden30	F	52	FTD	NA	Sporadic	UK	40
Snowden31	F	62	FTD	NA	Familial	UK	40
Snowden32	F	58	FTD	NA	Sporadic	UK	40
Soong1	F	46	ALS	Spinal	Familial	Taiwan	41
Soong2	F	55	ALS	Bulbar	Familial	Taiwan	41
Soong3	M	46	ALS	Spinal	Familial	Taiwan	41
Soong4	M	49	ALS	Spinal	Familial	Taiwan	41
Soong5	M	48	ALS	Spinal	Sporadic	Taiwan	41
Soong6	M	42	ALS	Bulbar	Sporadic	Taiwan	41
Ticozzi1	M	62	ALS	Bulbar	Familial	Italy	42
Ticozzi2	M	49	ALS	Spinal	Sporadic	Italy	42
Vrabec1	M	52	ALS	Spinal	Sporadic	Slovenia	43
Vrabec2	F	60	ALS	Spinal	Sporadic	Slovenia	43
Vrabec3	M	61	ALS-FTD	Spinal	Sporadic	Slovenia	43

Vrabec4	F	55	ALS	Bulbar	Sporadic	Slovenia	43
Vrabec5	M	70	ALS	Spinal	Sporadic	Slovenia	43

ALS = amyotrophic lateral sclerosis, FTD = frontotemporal dementia, F = female, M = male, NA = not available, UK = United Kingdom, US = United States

Supplemental References

1 Abramycheva, N. Y. *et al.* C9ORF72 hexanucleotide repeat expansion in ALS patients from the Central European Russia population. *Neurobiol. Aging* **36**, 2908 e2905-2909, doi:10.1016/j.neurobiolaging.2015.07.004 (2015).

2 Al-Sarraj, S. *et al.* p62 positive, TDP-43 negative, neuronal cytoplasmic and intranuclear inclusions in the cerebellum and hippocampus define the pathology of C9orf72-linked FTLD and MND/ALS. *Acta Neuropathol.* **122**, 691-702 (2011).

3 Alavi, A. *et al.* Repeat expansion in C9ORF72 is not a major cause of amyotrophic lateral sclerosis among Iranian patients. *Neurobiol. Aging* **35**, 267 e261-267, doi:10.1016/j.neurobiolaging.2013.07.016 (2014).

4 Beck, J. *et al.* Large C9orf72 hexanucleotide repeat expansions are seen in multiple neurodegenerative syndromes and are more frequent than expected in the UK population. *Am. J. Hum. Genet.* **92**, 345-353 (2013).

5 Benussi, L. *et al.* C9ORF72 hexanucleotide repeat number in frontotemporal lobar degeneration: a genotype-phenotype correlation study. *J. Alzheimers Dis.* **38**, 799-808 (2014).

6 Bouliotis, G. & Billingham, L. Crossing survival curves: alternatives to the log-rank test. *Trials* **12**, A137 (2011).

7 Cerami, C. *et al.* Novel evidence of phenotypical variability in the hexanucleotide repeat expansion in chromosome 9. *J. Alzheimers Dis.* **35**, 455-462 (2013).

8 Chester, C. *et al.* Rapidly progressive frontotemporal dementia and bulbar amyotrophic lateral sclerosis in Portuguese patients with C9orf72 mutation. *Amyotroph.Lat. Scl. Fr.* **14**, 70-72 (2013).

9 Chiò, A. *et al.* Clinical characteristics of patients with familial amyotrophic lateral sclerosis carrying the pathogenic GGGGCC hexanucleotide repeat expansion of C9ORF72. *Brain* **135**, 784-793 (2012).

10 Dobson-Stone, C. *et al.* C9ORF72 repeat expansion in clinical and neuropathologic frontotemporal dementia cohorts. *Neurology* **79**, 995-1001 (2012).

11 Dobson-Stone, C. *et al.* C9ORF72 repeat expansion in Australian and Spanish frontotemporal dementia patients. *PLoS One* **8**, e56899, doi:10.1371/journal.pone.0056899 (2013).

12 Dombroski, B. A. *et al.* C9orf72 hexanucleotide repeat expansion and Guam amyotrophic lateral sclerosis-Parkinsonism-dementia complex. *JAMA Neurol.* **70**, 742-745 (2013).

13 Ferrari, R. *et al.* Screening for C9ORF72 repeat expansion in FTLD. *Neurobiol. Aging* **33**, 1850.el-11, doi: 10.1016/j.neurobiolaging.2012.02.017 (2012).

14 Fratta, P. *et al.* Screening a UK amyotrophic lateral sclerosis cohort provides evidence of multiple origins of the C9orf72 expansion. *Neurobiol. Aging* **36**, 546 e541-547, doi:10.1016/j.neurobiolaging.2014.07.037 (2015).

15 Galimberti, D. *et al.* Incomplete penetrance of the C9ORF72 hexanucleotide repeat expansions: frequency in a cohort of geriatric non-demented subjects. *J. Alzheimers Dis.* **39**, 19-22 (2014).

16 Galimberti, D. *et al.* Autosomal dominant frontotemporal lobar degeneration due to the C9ORF72 hexanucleotide repeat expansion: late-onset psychotic clinical presentation. *Biol. Psychiatry* **74**, 384-391 (2013).

17 Gijselinck, I. *et al.* A C9orf72 promoter repeat expansion in a Flanders-Belgian cohort with disorders of the frontotemporal lobar degeneration-amyotrophic lateral sclerosis spectrum: a gene identification study. *Lancet Neurol.* **11**, 54-65 (2012).

18 Harms, M. *et al.* C9orf72 hexanucleotide repeat expansions in clinical Alzheimer disease. *JAMA Neurol.* **70**, 736-741 (2013).

19 Harms, M. B. *et al.* Lack of C9ORF72 coding mutations supports a gain of function for repeat expansions in amyotrophic lateral sclerosis. *Neurobiol. Aging* **34**, 2234.e2213-2239, doi:10.1016/j.neurobiolaging.2013.03.006 (2013).

20 Harms, M. B. *et al.* Parkinson disease is not associated with C9ORF72 repeat expansions. *Neurobiol. Aging* **34**, 1519.e1511-1512, doi:10.1016/j.neurobiolaging.2012.10.001 (2013).

21 He, J. *et al.* C9orf72 hexanucleotide repeat expansions in Chinese sporadic amyotrophic lateral sclerosis. *Neurobiol. Aging* **36**, 2660.e2661-2668, doi:10.1016/j.neurobiolaging.2015.06.002 (2015).

22 Ishiura, H. *et al.* C9ORF72 repeat expansion in amyotrophic lateral sclerosis in the Kii peninsula of Japan. *Arch. Neurol.* **69**, 1154-1158 (2012).

23 Jiao, B. *et al.* Identification of C9orf72 repeat expansions in patients with amyotrophic lateral sclerosis and frontotemporal dementia in mainland China. *Neurobiol. Aging* **35**, 936.e919-922, doi:10.1016/j.neurobiolaging.2013.10.001 (2014).

24 Kaivorinne, A. L. *et al.* Clinical Characteristics of C9ORF72-Linked Frontotemporal Lobar Degeneration. *Dement. Geriatr. Cogn. Dis. Extra* **3**, 251-262 (2013).

25 Kandiah, N. *et al.* Rapidly Progressive Dementia in a Chinese Patient due to C9ORF72 Mutation. *Can. J. Neurol. Sci.***39**, 676-677 (2014).

26 Kenna, K. P. *et al.* Delineating the genetic heterogeneity of ALS using targeted high-throughput sequencing. *J. Med. Genet.* **50**, 776-783 (2013).

27 Konno, T. *et al.* Japanese amyotrophic lateral sclerosis patients with GGGGCC hexanucleotide repeat expansion in C9ORF72. *J. Neurol. Neurosurg. Psychiatry* **84**, 398-401 (2012).

28 Lindquist, S. G. *et al.* Corticobasal and ataxia syndromes widen the spectrum of C9ORF72 hexanucleotide expansion disease. *Clin. Genet.* **83**, 279-283 (2013).

29 Majounie, E. *et al.* Repeat expansion in C9ORF72 in Alzheimer's disease. *N. Engl. J. Med.* **366**, 283-284 (2012).

30 Mandic-Stojmenovic, G. *et al.* Screening for C9orf72 Expansion Mutation in Serbian Patients with Early-Onset Dementia. *Dement. Geriatr. Cogn. Dis.* **40** (2015).

31 Millecamps, S. *et al.* Phenotype difference between ALS patients with expanded repeats in C9ORF72 and patients with mutations in other ALS-related genes. *J. Med. Genet.* **49**, 258-263 (2012).

32 Mok, K. Y. *et al.* High frequency of the expanded C9ORF72 hexanucleotide repeat in familial and sporadic Greek ALS patients. *Neurobiol. Aging* **33**, 1851.e1851-1855, doi:10.1016/j.neurobiolaging.2012.02.021 (2012).

33 Murray, M. E. *et al.* Clinical and neuropathologic heterogeneity of c9FTD/ALS associated with hexanucleotide repeat expansion in C9ORF72. *Acta Neuropathol.* **122**, 673-690 (2011).

34 Ogaki, K. *et al.* Analysis of C9orf72 repeat expansion in 563 Japanese patients with amyotrophic lateral sclerosis. *Neurobiol. Aging* **33**, 2527.e2511-2526, doi:10.1016/j.neurobiolaging.2012.05.011 (2012).

35 Ozoguz, A. *et al.* The distinct genetic pattern of ALS in Turkey and novel mutations. *Neurobiol. Aging* **36**, 1764.e1769-1718, doi:10.1016/j.neurobiolaging.2014.12.032 (2015).

36 Pamphlett, R., Cheong, P. L., Trent, R. J. & Yu, B. Transmission of C9orf72 hexanucleotide repeat expansions in sporadic amyotrophic lateral sclerosis: an Australian trio study. *Neuroreport* **23**, 556-559 (2012).

37 Sabatelli, M. *et al.* C9ORF72 hexanucleotide repeat expansions in the Italian sporadic ALS population. *Neurobiol. Aging* **33**, 1848.e15-20, doi: 10.1016/j.neurobiolaging.2012.02.011 (2012).

38 Snowden, J. S. *et al.* Distinct clinical and pathological characteristics of frontotemporal dementia associated with C9ORF72 mutations. *Brain* **135**, 693-708 (2012).

39 Soong, B. W. *et al.* Extensive molecular genetic survey of Taiwanese patients with amyotrophic lateral sclerosis. *Neurobiol. Aging* **35**, 2423.e2421-2426, doi:10.1016/j.neurobiolaging.2014.05.008 (2014).

40 Ticozzi, N. *et al.* C9orf72 repeat expansions are restricted to the ALS-FTD spectrum. *Neurobiol. Aging* **35**, 936.e913-937, doi:10.1016/j.neurobiolaging.2013.09.037 (2014).

41 Vrabec, K. *et al.* Genetic analysis of amyotrophic lateral sclerosis in the Slovenian population. *Neurobiol. Aging* **36**, 1601.e1617-1620, doi:10.1016/j.neurobiolaging.2014.11.011 (2015).
